# Spintronic leaky-integrate-fire spiking neurons with self-reset and winner-takes-all for neuromorphic computing

**DOI:** 10.1038/s41467-023-36728-1

**Published:** 2023-02-24

**Authors:** Di Wang, Ruifeng Tang, Huai Lin, Long Liu, Nuo Xu, Yan Sun, Xuefeng Zhao, Ziwei Wang, Dandan Wang, Zhihong Mai, Yongjian Zhou, Nan Gao, Cheng Song, Lijun Zhu, Tom Wu, Ming Liu, Guozhong Xing

**Affiliations:** 1 Key Laboratory of Microelectronics Devices & Integration Technology, Institute of Microelectronics, Chinese Academy of Sciences, 100029 Beijing, China; 2 University of Chinese Academy of Sciences, 100049 Beijing, China; 3 Department of Electrical Engineering and Computer Sciences, University of California, Berkeley, CA 94720 USA; 4 Institute of Metal Research, Chinese Academy of Sciences, Shenyang, 110016 China; 5 School of Microelectronics, University of Science and Technology of China, Hefei, 230026 Anhui China; 6 Jiufengshan Laboratory, Wuhan, 430206 Hubei China; 7 Key Laboratory of Advanced Materials (MOE), School of Materials Science and Engineering, Tsinghua University, 100084 Beijing, China; 8 State Key Laboratory of Superlattices and Microstructures, Institute of Semiconductors, Chinese Academy of Sciences, 100083 Beijing, China; 9 Department of Applied Physics, The Hong Kong Polytechnic University, Kowloon, Hong Kong, China

**Keywords:** Electrical and electronic engineering, Electronic and spintronic devices

## Abstract

Neuromorphic computing using nonvolatile memories is expected to tackle the memory wall and energy efficiency bottleneck in the von Neumann system and to mitigate the stagnation of Moore’s law. However, an ideal artificial neuron possessing bio-inspired behaviors as exemplified by the requisite leaky-integrate-fire and self-reset (LIFT) functionalities within a single device is still lacking. Here, we report a new type of spiking neuron with LIFT characteristics by manipulating the magnetic domain wall motion in a synthetic antiferromagnetic (SAF) heterostructure. We validate the mechanism of Joule heating modulated competition between the Ruderman–Kittel–Kasuya–Yosida interaction and the built-in field in the SAF device, enabling it with a firing rate up to 17 MHz and energy consumption of 486 fJ/spike. A spiking neuron circuit is implemented with a latency of 170 ps and power consumption of 90.99 μW. Moreover, the winner-takes-all is executed with a current ratio >10^4^ between activated and inhibited neurons. We further establish a two-layer spiking neural network based on the developed spintronic LIFT neurons. The architecture achieves 88.5% accuracy on the handwritten digit database benchmark. Our studies corroborate the circuit compatibility of the spintronic neurons and their great potential in the field of intelligent devices and neuromorphic computing.

## Abstract (web summary)

Designing bio-inspired artificial neurons within a single device is challenging. Here, the authors demonstrate a spintronic neuron with leaky-integrate-fire and self-reset characteristics and corroborate a new trajectory of all-spin neuromorphic computing hardware holistic implementation.

## Introduction

Neuromorphic computing (NC) imitates the functions of the brain by utilizing a network of synthetic neurons interconnected among synaptic devices^1,2^. Owing to its potential for artificial intelligence (AI) and big data analysis in an energy-efficient manner beyond the traditional von Neumann computing system, NC is attracting intensive attention worldwide and promising to deliver increased intelligence for autonomous driving, embedded artificial intelligence of things (AIoT) and terminal devices^1–5^. Since early 2000s researchers found that it is feasible to develop neuromorphic neuron and synapse devices to realize complex and highly reliable neural networks on a chip^6^, there have been many attempts to simulate the brain’s functions using traditional silicon technology over the last two decades. But AI is asking questions about the best way to build an NC system. Researchers have been trying to mimic the various characteristics of biological neurons^7^ utilizing either traditional complementary metal-oxide-semiconductor (CMOS) technology^8^ or emerging nonvolatile memory (NVM) devices, such as spintronic memory^9–14^, resistive switching memory^15,16^, phase change memory^17^, and ferroelectric memory^18^. However, most of these approaches, especially traditional CMOS technology-based neuron circuits, require multiple capacitors and dozens of transistors, which involve enormous amounts of power and area for emulating complex behaviors of biological neurons^1^.

In contrast, the NC based on NVMs promises to provide a more efficient solution for complex tasks such as pattern recognition, machine learning and edge computing, as they can better simulate the biological characteristics of neurons, e.g., leaking, integrating, firing and auto-reset capabilities with less or free of transistor and capacitors^1,2^. Among them, spintronic NVM, which allows the implementation of nonlinear magnetization dynamics on nanoscale, provides numerous opportunities in this field^3,19^. In the research community of spintronic neuron devices, the studies of magnetic skyrmion and domain wall (DW) are rising^20^. However, the injection and manipulation of skyrmion are still immature^21^ and the skyrmion-based devices require exotic or wedge-shaped structures^12,13^, rendering the deteriorated stability and high process complexity. In contrast, magnetic DW nucleation and manipulation techniques are intensively developed^22–24^. The DW-based spintronic devices combine unique features that other technologies cannot match, including non-volatility, outstanding read/write endurance, high-speed operation, and high scalability. Nevertheless, the reported DW-neuromorphic devices warrant considerable improvements. For example, Fan et al.^14^ reported a non-linear spin-transfer torque neuron (STT-Neuron) with neuron circuit application but it lacks the leaky characteristics like biological neurons, in particular, the essential reset operation inevitably involves the complex operation and increased power consumption. Hassan et al.^11^ demonstrated a type of DW-Neuron with leaky-integrate-fire (LIF) characteristics by applying a large area of the hard magnetic layer and claimed a local inhibition between adjacent neurons through the stray field between devices. However, these reports are purely based on the simulation framework with no genuine global inhibition, hence it is urgent to experimentally verify the feasibility of DW based LIF devices with self-reset and complementary device-circuit implementation with superior power efficiency to conventional computers, to facilitate the integrated AI applications in CMOS compatible industrial level mass production.

Moreover, it is expected to solve reliability, area cost and energy efficacy bottlenecks and provide more possibilities for learning and computing processes of large-scale NC. Typically, the magnetoresistive random-access memory (MRAM) bears competitive advantages of high read and write speed, high reliability, ultra-low power consumption, and nearly infinite endurace^25,26^. Therefore, developing neuromorphic hardware based on the advanced MRAM components is paramount important from the practical application perspectives, in which the synthetic antiferromagnet (SAF)^27–29^ poises as the kernel of the commercialized MRAM cell, i.e., magnetic tunnel junction (MTJ) as described in early reports and our previous works^3,30–38^.

In the present work, to tackle the aforementioned challenges and bottleneck, upon extensive investigations on the dynamic process of field- and current-driven DW motion (DWM) in the SAF heterostructure, we further explore the modulation of interlayer-exchange-coupling (IEC) by engineering the dynamic Joule heating. Importantly, for the first time, we propose a new type of highly reliable spintronic neuron device with leaky-integrate-fire and self-reset (LIFT) features based on the tailored DWM in the spin-polarized ferro-coupler layers of SAF heterostructure, intrinsically mimicking the LIFT behaviors of neurons under a synergistic effect of built-in field (H_built-in_) and Ruderman–Kittel–Kasuya–Yosida (RKKY) interaction without any additional reset devices or circuitry. Aiming at the CMOS compatible and manufacturable application, the winner-takes-all (WTA)^39^ with global inhibition has been realized among the developed neuron devices by differentiating current through the negative differential resistance (NDR) effect of the semiconductor^40^, and the feasibility of the neuron device in the spiking neural networks (SNN) and its good circuit compatibility with high performance were verified.

## Results

### SAF characteristics and DWM dynamics

As a representative magnetic soliton^41^, the magnetic DWs can be reliably driven by field and spin-polarized currents. However, the method in which the DWs are driven by an external field is very inconvenient and not conducive to the CMOS compatible integration application. Therefore, it is necessary to construct an intrinsic built-in effective field in a well-established magnetic heterostructure that can be widely utilized in commercialized products. At present, in addition to the deposition of hard magnet layer^42^ and exchange bias interaction^43^, the IEC from RKKY interaction in a developed and matured SAF device structure^44^ is indeed an effective scheme.

**Fig. 1:**
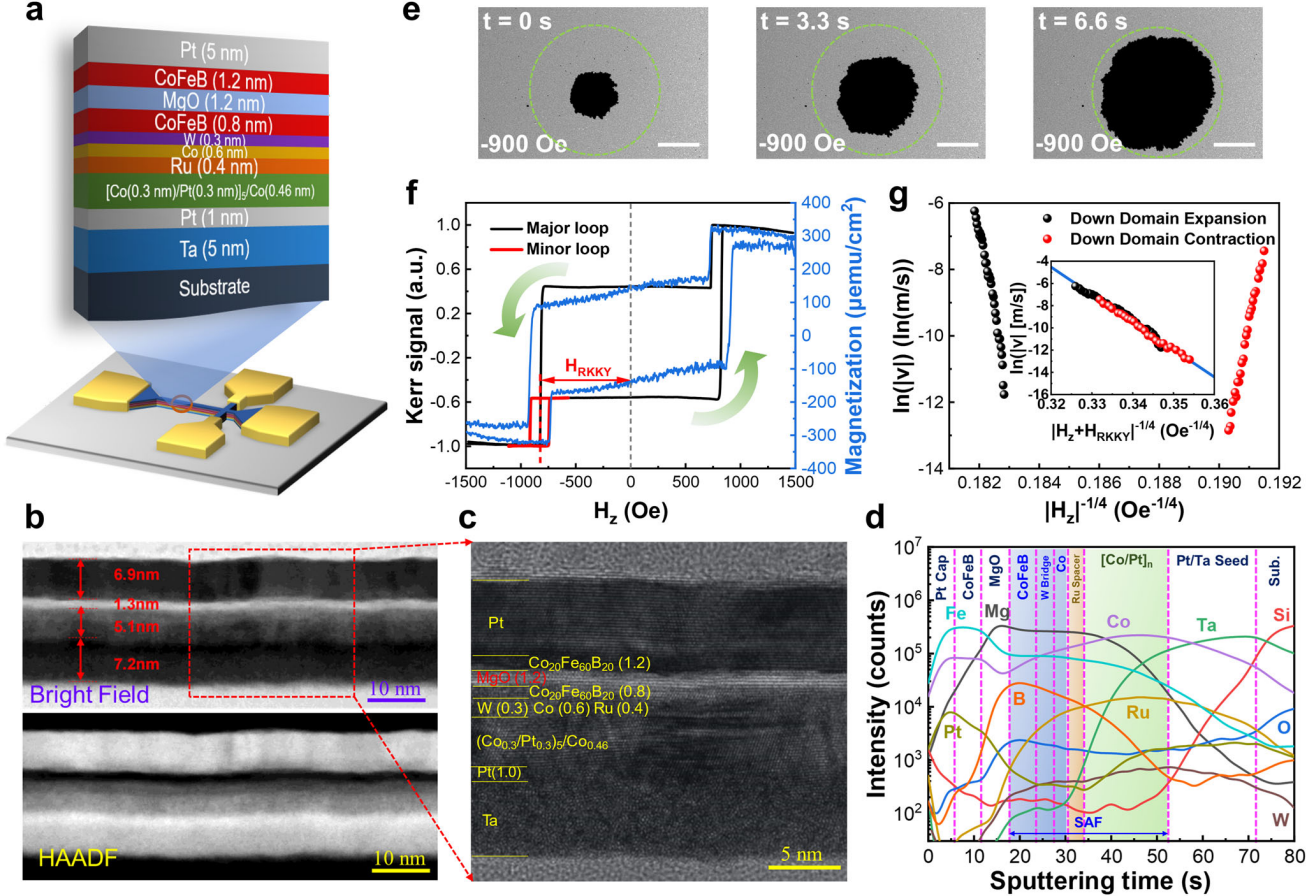
Domain wall motion dynamics in SAF structure. **a** Schematic of films stack and patterned device. Corresponding STEM and HAADF **b**, HRTEM **c** images and atomic level composition depth profile using SIMS analysis **d**. **e** Kerr images recorded at different time under an OOP magnetic field of 900 Oe. The black and gray region refers to down and up domain, respectively. DW configuration is marked by green dashed circles. The scale bar is 500 μm. **f** Major K-H loop of the multilayered films sample. H_RKKY_ denotes effective field from RKKY interaction. **g** Relationship between ln|v| and |H_z_| during expansion and contraction processes. Inset shows the ln|v| data against |H_z_ + H_RKKY_|^−1/4^ with H_RKKY_ of 826 Oe from RKKY interaction.

In our experiments, the thin-film samples of substrate/Ta(5)/Pt(1)/[Co(0.3)/Pt(0.3)]_5_/Co(0.46)/Ru(0.4)/Co(0.6)/W(0.3)/CoFeB(0.8)/MgO(1.2)/CoFeB(1.2)/Pt(5) were deposited by a magnetron DC/RF sputtering tool, the thickness of every layer in nanometers is indicated in parentheses, as shown in Fig. 1a. The high-angle annular dark-field (HAADF) image in Fig. 1b indicates smooth interfaces and high crystalline quality in the stacks. The epitaxial growth of individual layers with clear interfaces was revealed by high-resolution bright-field scanning transmission electron microscope (STEM). The high-resolution TEM (HRTEM) image in Fig. 1c indicates that a virtually perfect single-crystalline and continuous film stack is obtained with specifically defined thickness. The distribution of elements and the minimized intermixing at the interfaces in the films stack were analyzed by secondary electron mass spectrometry (SIMS), as shown in Fig. 1d. The correct and uniform distribution of Co, Pt and Fe atoms in SAF is verified and supports the high quality of the film and the good perpendicular magnetic anisotropy (PMA) in the perpendicularly spin-polarized (PSP) layer of CoFeB(0.8) ferromagnetically coupled with Co(0.6) (Fig. 1f).

First, the DW motion driven by the effective IEC field in the PSP ferromagnetic layer of the SAF structure was studied in detail. The DW velocity measurements were performed by applying a positive field saturation pulse and an image for reference under a small negative bias field was recorded. A short negative field pulse was then applied to nucleate DW, followed by a constant negative/positive field to drive domain expansion/contraction, during which a polar magneto-optic Kerr effect (p-MOKE) microscope kept recording the Kerr images. The DW velocity can be determined from the domain expansion/contraction displacement and the corresponding interval time. The Kerr images of DW motion in a single ferromagnetic layer under the external field and IEC field are shown in Fig. 1e. The major loop in Fig. 1f exhibits PMA from the PSP layer of CoFeB(0.8) coupled with the upper layer Co(0.6) in SAF, and the minor loop of the PSP layer was measured after the hard layer was negatively saturated. As a result, a minor loop shift of −826 Oe was observed, implying an effective IEC field of 826 Oe, which can be expressed as^28^,

1 $${{{{{{\rm{H}}}}}}}_{RKKY}=\frac{{J}_{RKKY}}{{M}_{s}t}$$

where H_*RKKY*_ denotes the effective IEC field, and *J*_*RKKY*_ is the IEC energy density. *M*_*s*_ and *t* are the saturation magnetization and thickness of the ferromagnetic layer.

A natural logarithm of DW velocity against the external field was plotted and an asymmetry of the relationship between velocity and field during down domain expansion and contraction was observed, as shown in Fig. 1g. In contrast, an obvious linear relationship between ln|v| and |H_z_ + H_RKKY_|^−1/4^ (inset of Fig. 1g), satisfies the creep law^45^ strictly and shows that the DW dynamics of expansion and contraction comply with the creep law and both are identical. This further indicates that the IEC is equivalent to an out-of-plane (OOP) effective field of 826 Oe, which drives the DW motion. To gain deeper insights into the DW motion dynamics under modulation by H_x_^46^, a series of SAF heterostructures were grown with a dedicated in-plane magnetic anisotropic (IMA) CoFeB(1.2) layer on the top of MgO, as illustrated in Fig. 1a. The specific growth and post-processing conditions can be found in our previous report^18^. In this work, the samples with moderate IMA in the top layer of CoFeB(1.2) were selected, as demonstrated by the symmetry expansion of DW and the hysteresis double-loop (Fig. 1e, f). On one hand, the influence of ambient field on DW motion in the PSP layer of SAF can be eliminated with magnetic immune protection from the top IMA CoFeB. On the other hand, the tilted magnetic anisotropy may also provide an additional regulation knob to tune the DW motion velocity with different easy axis angles relative to the normal direction.

### Spintronic neurons with LIFT characteristics

**Fig. 2:**
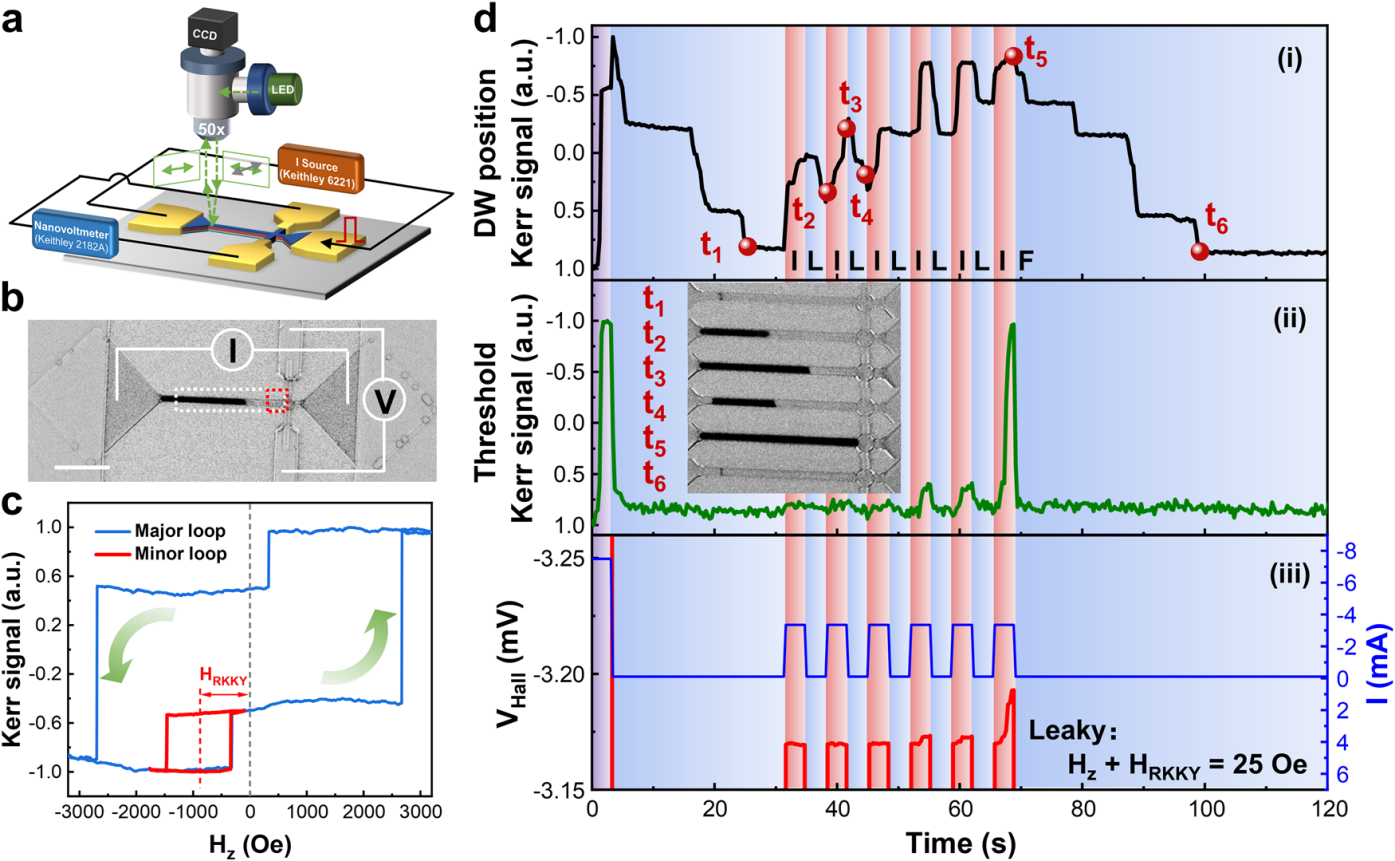
Prototype spintronic neuron device with LIF characteristics. **a** Schematic of p-MOKE setup for in-situ magneto-electrical transport probing. **b** Kerr image with the yellow dashed box indicating DW stripe and Kerr signal in **d**(i), red dashed box refers to the threshold region and corresponding Kerr signal in **d**(ii). The scale bar is 20 μm. **c** Major K-H Loop of the device. H_RKKY_ refers to effective field from RKKY interaction and is equaled to 885 Oe. **d** Dynamic DW motion emulated LIF processes under an applied |H_z_| field that partially offsets H_RKKY_ with an effective net field of ~25 Oe. Joule heating modulated RKKY, dynamic DW motion processes are recorded at different stage as depicted in (i), the reversal characteristics of threshold region in (ii), and anomalous Hall voltage under associated applied current pulses in (iii).

A series of Hall bar-like devices were fabricated and the corresponding Kerr images were recorded as shown in Fig. 2a, b. From Fig. 2c, an obvious shift from measured major and minor Kerr magnetic hysteresis loops validates that there is an effective field of 885 Oe existing between the ferro-coupled layers of CoFeB/Co and the bottom hard layer in the SAF heterostructure. Both enhanced coercivity and RKKY effective fields are attributed to the device shrinking^47^ and the unavoidable peripheral edge damages introduced during the ion beam etching (IBE) process^48^. Due to the high IEC existence in our samples, a constant external OOP field of −860 Oe was applied to compensate for the RKKY field in the experiments. Briefly, a 3 s pulsed current of 7.5 mA was firstly injected along the ˗*x* direction of the Hall bar and a nucleated down domain was observed at the left end of the strip with a favored expansion to the +*x* region. Subsequently, upon a RKKY equivalent field application, both DWs on the left and right sides contracted towards the nucleation point center, i.e., the DW contraction. Then, a 50% duty cycle pulsed current of 3.35 mA was injected into the strip from the *x* direction for six consecutive 6 s cycles, and the down-up (DU) DW gradually moved to the right driven by the current, while it moved to the left under the RKKY interaction when the current was removed. After the sixth pulse was applied, the DU DW reached the cross region of the Hall bar, and a Hall voltage signal was sensed through the anomalous Hall effect (AHE) as an output signal. Such current input-voltage output scheme is explicitly compatible with the in-memory neuromorphic computing architecture. It is noted that the DU DW fell back to the initial position with typical self-reset manners of an ideal neuron under the net effective RKKY field. The aforementioned DW motion dynamic processes biologically emulate the integration, leakage, and firing of ideal neurons. As to a brief ‘refractory’ period after firing, the neuron device will not integrate any input spike current. The complete process is revealed in Fig. 2d and Supplementary Movie 1, and the corresponding state of DW during the process is illustrated in the insets, respectively. The appearance of DW pinning during the integration and leakage is caused by randomly distributed pinning centers^49^. Note that to alleviate the DW motion stochasticity issue in our work, we employed the synergistic co-optimization approach from material physics, device fabrication process engineering and LIFT neuron devices’ operation mechanism (Supplementary Note S1).

### RKKY interaction modulation by Joule heating

**Fig. 3:**
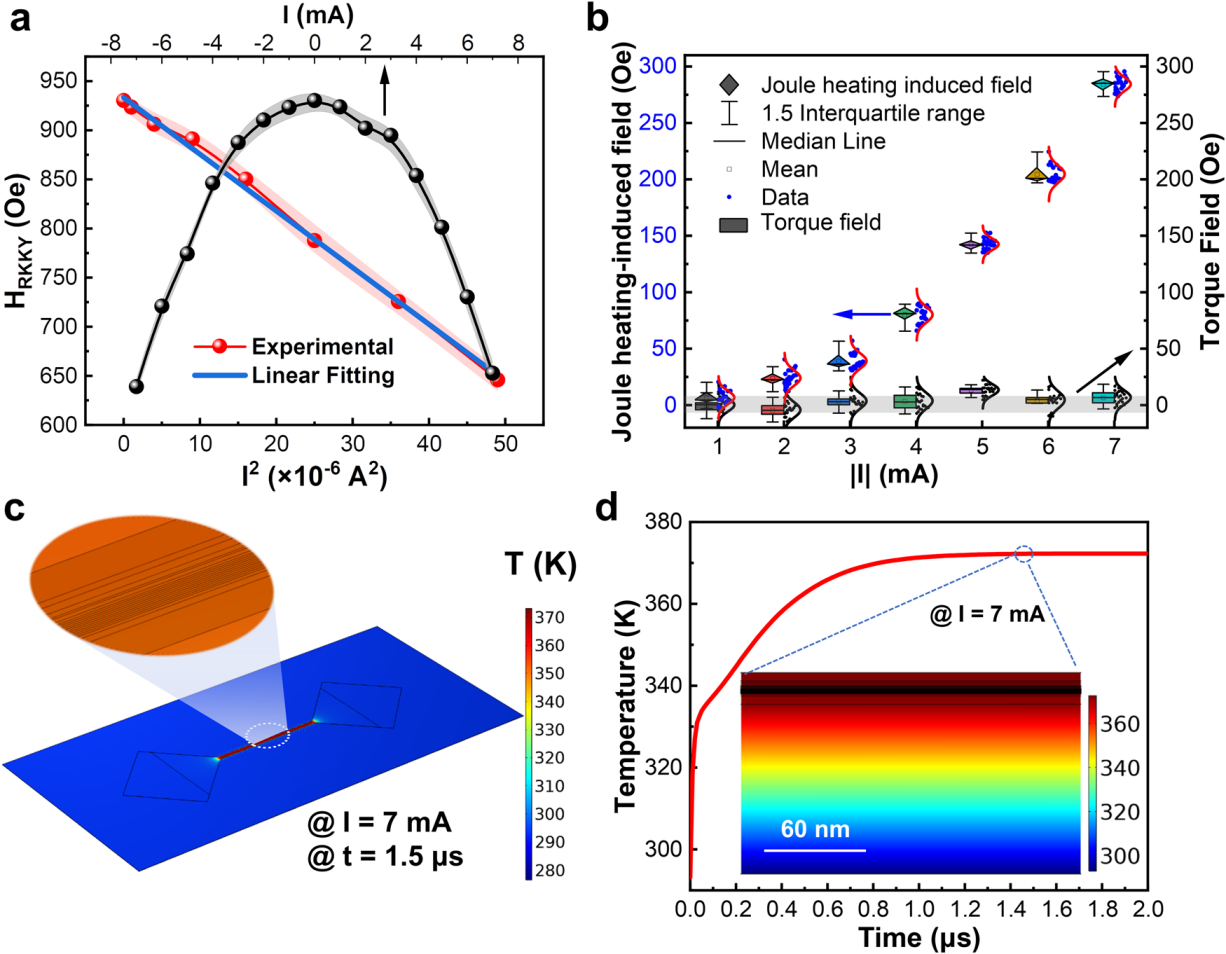
Modulated RKKY and time- and current-dependent temperature effects. **a** RKKY effective field as a function of the current with RKKY effective field *vs* I^2^. The bright red shaded areas represent error bars. **b** The dominant Joule heating-induced field (JHIF) with a weak torque field. **c** Simulated temperature distribution of device with the same dimension as the experiments. **d** Time-dependent temperature at the center of device as highlighted in the white dashed circle of **c**. Inset illustrates the cross-section of temperature distribution profile along the length of device.

It is found that the direction of DW motion was independent of current polarity, which suggests that the driving force is neither solely the STT nor spin-orbit torque (SOT). In contrast, our extensive experimental investigations corroborate that the RKKY effective field is primarily modulated by Joule heating with the negligible field via STT and/or SOT effects. Specifically, in addition to the serious shunting effect in such a SAF film stack, STT generated by the in-plane current that is originated from the ferromagnetic layer itself, which usually has low spin polarization, especially in metal multilayers, the effective spin-polarized carriers will be further reduced due to the non-trivial spin scattering in different layers^50^, while SOT usually shows appreciable spin Hall effect only when the thickness of heavy metal is greater than the spin diffusion length^51^. The dynamic DW motion detected is plausibly attributed to the competition between the RKKY effective field and the built-in interlayer-coupled field in SAF. Briefly, when the RKKY effective field is modulated via Joule heating generated by pulsed current^52^, both amplitude and polarity of the effective field acting on DW are changed, thus driving the reciprocating motion of DW. As shown in Fig. 3a, the RKKY effective field varies as a function of applied current under an ambient environment. The current bears a significant regulation effect on the RKKY effective field with a parabolic correlation. Furthermore, it was found that the RKKY effective field is linearly dependent on the square of current consolidating that the Joule heating generated by current plays an intuitive and critical role in tailoring the RKKY interaction. As further manifested in Fig. 3b, the non-Joule-heating-induced torque field strength is less than 5 Oe, which is <10% of the Joule heating-induced-field (JHIF). Clearly, the JHIF is dominated in the modulation of the RKKY interaction, corroborated by our reproducible experiments (for more details, refer to Supplementary Note S2). This approach is also technically in line with the report on the narrow heater bottom electrode-based memory as a bidirectional artificial synapse^53^.

To quantitatively analyze the Joule heating distribution and associated temperature evolution in our devices, the COMSOL multiphysics, i.e., a cross-platform finite element analysis tool was employed to simulate the Joule heating effect. The basic parameters utilized are listed in Supplementary Table S1. Based on the actual film stack materials and device dimension, a dedicated model was established by applying a pulsed current of 7 mA for 2 μs, which aligns with the experimental conditions. As shown in Fig. 3c, the temperature of the device increases up to 372 K. Note that the center temperature in SAF saturates at 1.5 μs under ideal boundary constrained conditions, as illustrated in Fig. 3d. The inset shows the temperature distribution at the center of the device along an approximately 200 nm section. By combining the experimental and simulation results, the estimated change rate of the RKKY effective field with temperature is about 3.6 Oe/K, which is consistent with the previous reports^54,55^. To examine the temperature impact on RKKY interaction in the subsequent works, the correlation between the RKKY field and temperature abides by the linear dependence strictly, i.e., the RKKY interaction change rate is 0.18%/K. The above results motivate us to further explore and utilize the competition between RKKY interaction and built-in field, which can be generated by a hard ferromagnet and or so, in turn, to realize an all-electrical control spintronic neuron device.

### Optimization and scaling of spintronic neuron devices

**Fig. 4:**
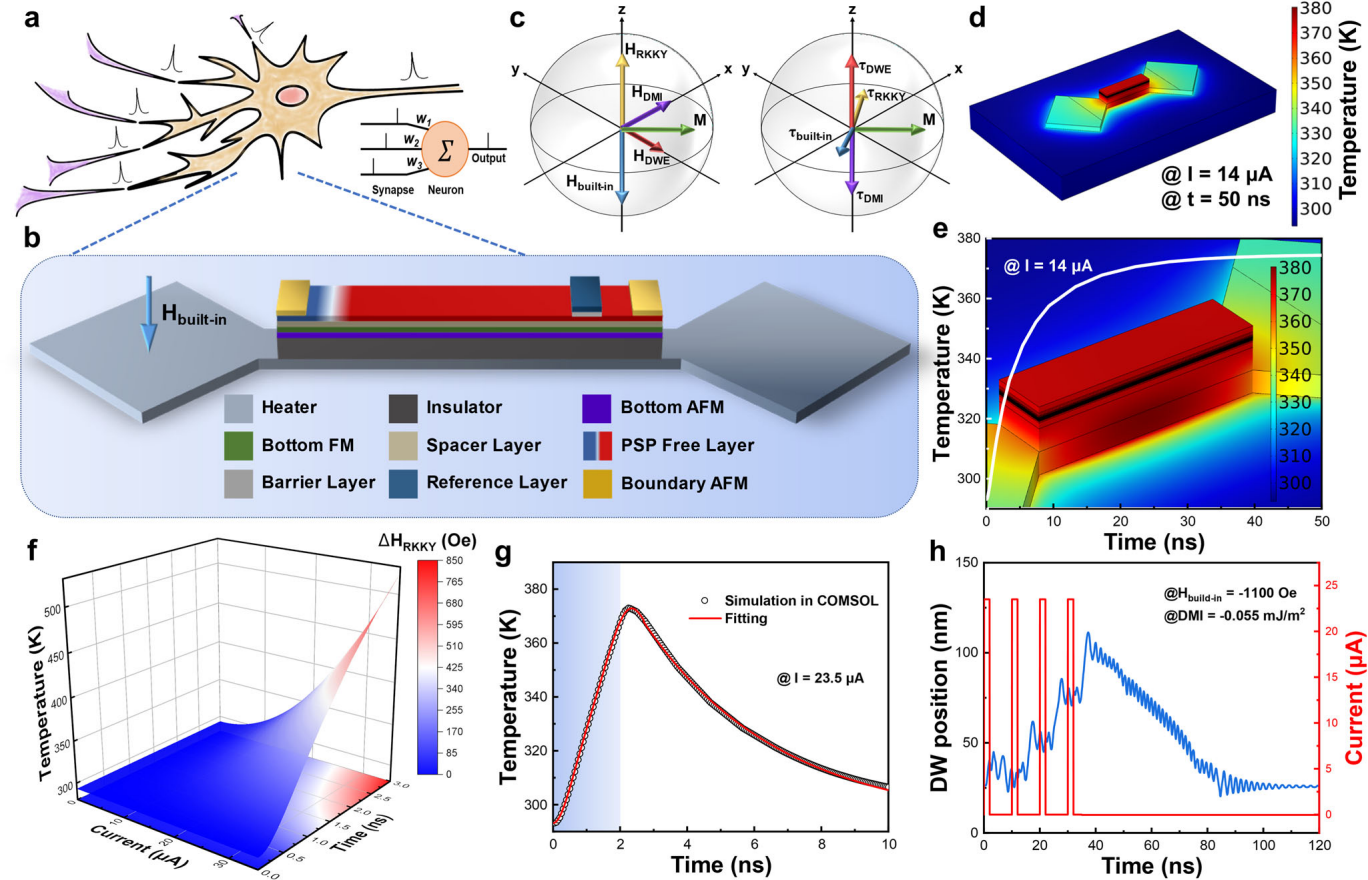
Joule heating temperature and DWM characteristics in the proposed device. Schematic of a biological neuron **a** and proposed spintronic neuron **b**. **c** Schematic directions of effective fields and torques of upper ferromagnetic layer. M denotes the magnetization in DW center, the longitudinal torque τ_DMI_, exchange torque τ_RKKY_, DW energy torque τ_DWE_ and built-in torque τ_built-in_ is derived from the longitudinal field H_DMI_, antiferromagnetic exchange coupling field H_RKKY_, DW energy fields H_DWE_ and H_built-in_, respectively. Static **d** and dynamic **e** temperature distribution of device under 14 μA DC current. **f** Phase diagram of temperature and the modulated RKKY effective field evolution. **g** Dynamic temperature upon 23.5 μA and 2 ns pulsed current. **h** DW LIFT characteristics.

The human nervous system contains about 10^11^ neurons and 10^15^ synapses^56^. As schematically illustrated in Fig. 4a, the pre-neuron signals can be transmitted to the post-neuron via synaptic weighting. Remarkably, some typical models have been developed to mimic the characteristics of neurons. Among various neuronal models, the LIF neuron model^7,12^ is widely accepted as it can better mimic the characteristics of biological neurons with the minimum number of circuit elements, unlike other models. In addition to emulating the process by which a neuron will fire only when the input signal exceeds a threshold, it also can fully portray the leakage nature of a neuron.

To further enhance the energy and time efficiency, a 20 nm thick semiconductor ScN with appropriate resistivity^57^ is utilized as a heater, and the length and width of the device are scaled down to 220 nm and 50 nm, respectively. The proposed neuron device consists of individual key layer schematically as shown in Fig. 4b. The upper ferromagnetic layer of the SAF multilayer serves as the PSP free layer (FL), while the lower one is pinned in the opposite magnetization to the FL by the bottom antiferromagnetic pinning layer. These two layers form the antiferromagnetic coupling through the spacer layer with the RKKY interaction. An MTJ is used for detecting and releasing signals with a higher sensing margin. The magnetization orientation of the RL is preset as downward. The bottom antiferromagnetic layer is pinned towards the +z direction so that the magnetization of the bottom ferromagnetic layer in SAF can be pinned along the -z direction. Moreover, upon RKKY antiferromagnetic coupling interaction, the PSP FL is always exerted by a positive effective field. However, the leftmost region of the FL will be pinned in the -z direction by the left antiferromagnetic pinning layer with +z direction under a preset external magnetic field. Therefore, the configuration of a DU DW is generated near the boundary of the left antiferromagnetic pinning region. In addition, the right antiferromagnetic pinning layer possesses an opposite magnetization direction to the left so that the DW can move in the range between two terminal antiferromagnetic pinning regions without annihilation.

DW motion dynamics is governed by the Landau–Lifshitz–Gilbert (LLG) equation^58^,

2 $$\frac{d{\hat{{{{{\mathbf{m}}}}}}}}{dt}=-\gamma \hat{{{{{{\mathbf{m}}}}}}}\times {{{{{\mathbf{H}}}}}}_{{{e}}{{f}}{{f}}}+\alpha \hat{{{{{{\mathbf{m}}}}}}}\times \frac{d\hat{{{{{{\mathbf{m}}}}}}}}{dt}$$

where *γ* is the gyromagnetic ratio, *α* is the Gilbert damping constant, and $\hat{{{{{{\mathbf{m}}}}}}}$ is the unit vector along the magnetization of the FM. With the Dzyaloshinskii–Moriya interaction (DMI)^59^ at the HM/FM interface in SAF, the effective magnetic field ${{{{{\mathbf{H}}}}}}_{eff}$ can be written as^58^,

3 $${{{{{\mathbf{H}}}}}}_{{{e}}{{f}}{{f}}}=	 \frac{2A_{ex}}{M_{s}}\frac{{\partial }^{2}\hat{{{{{{\mathbf{m}}}}}}}}{\partial {x}^{2}}+\frac{2K}{M_{s}}m_{z}\hat{{{{{{\mathbf{z}}}}}}}+\frac{2K_{d}}{M_{s}}m_{y}\hat{{{{{{\mathbf{y}}}}}}}-\frac{2D_{0}}{M_{s}}\left(\hat{{{{{{\mathbf{y}}}}}}}\times \frac{\partial \hat{{{{{{\mathbf{m}}}}}}}}{\partial x}\right)\\ 	+{{{{{{\rm{H}}}}}}}_{{{{{{\rm{R}}}}}}{{{{{\rm{K}}}}}}{{{{{\rm{K}}}}}}{{{{{\rm{Y}}}}}}}+{{{{{{\rm{H}}}}}}}_{{{{{{\rm{b}}}}}}{{{{{\rm{u}}}}}}{{{{{\rm{i}}}}}}{{{{{\rm{l}}}}}}{{{{{\rm{t}}}}}}-{{{{{\rm{i}}}}}}{{{{{\rm{n}}}}}}}$$

where *A*_*ex*_ is the exchange stiffness constant, *K*_*d*_ refers to the hard-axis anisotropy and *D*_*0*_ denotes the DMI constant.

A modified RKKY exchange field term evaluated using a 6-neighbor small-angle approximation is included in the effective field^60^,

4 $${{{{{{\boldsymbol{H}}}}}}}_{{{{{{\rm{RKKY}}}}}}}=2\frac{{A}_{{{{{{\rm{RKKY}}}}}}}}{{M}_{s}}\mathop{\sum}\limits_{i}\frac{({\hat{{{{{{\boldsymbol{m}}}}}}}}_{i}-\hat{{{{{{\boldsymbol{m}}}}}}})}{{\varDelta }_{i}^{2}}$$

where *A*_*RKKY*_ is the RKKY exchange stiffness constant, *M*_*s*_ is the saturation magnetization, and Δ_*i*_ is the thickness of the ferromagnetic cell. The fields and torques acting on the magnetic moments at the center of DW are shown in Fig. 4c and Supplementary Fig. S2. The temperature-dependent RKKY modulation is attributed to the weakened IEC due to the softening of the Fermi edge at higher temperatures and the complex reflection coefficients at the spacer/magnet interface^52^.

In the nanoscale devices with a heater, the device can be heated up to the same level as that in the experiment by using a current of two orders lower in magnitude, as shown in Fig. 4d. And it takes only 50 ns for the temperature to saturate, as depicted in Fig. 4e. Figure 4f demonstrates a phase diagram of temperature evolution with current amplitude and time, and the corresponding variation of RKKY effective field strength is mapped in color contour based on the change rate obtained from our experiments. Therefore, the energy and time costs can be optimized by compromising current amplitude and pulse width. As shown in Fig. 4g, with a pulsed current of 23.5 μA in 2 ns, one can achieve the desired temperature at the ns time scale. Based on the temperature rising and dissipation evolution diagram, a simple mathematical model can be fitted and introduced into MuMax3 for micromagnetic simulations^60^. Briefly, when four current pulses were applied with a width of 2 ns and a period of 10 ns, the RKKY field exertion on DWM was effectively and precisely reduced through the Joule heating generated by the heater. When the net effect of competition between RKKY interaction and the built-in field presents a negative effective field, DW begins to move to the right. When the current is removed, the temperature begins to dissipate, and the RKKY interaction gradually increases. When the net effect of the RKKY interaction and the built-in field present a positive effective field, DW begins to move to the left. The process of neuron integration and auto-leakage is reliably executed in a commercialized SAF device by repeating the above operations, as demonstrated in Fig. 4h and Supplementary Movie 2.

The comparison among different neuron devices (Supplementary Table S3) with calculated power and energy consumption (Supplementary Note S3) corroborates that the developed spintronic neuron devices successfully mimicked the LIFT characteristics of biological neurons. The rising time of 10 ns and the falling time of 50 ns further warrant the application of high-speed NC. Although the energy consumption of the neuron device is about 486 fJ/spike, it is still applicable to gradually approach or even surpass the energy consumption of biological neurons through structural minimization and Joule heating optimization.

### WTA spiking neuron circuit implementation

**Fig. 5:**
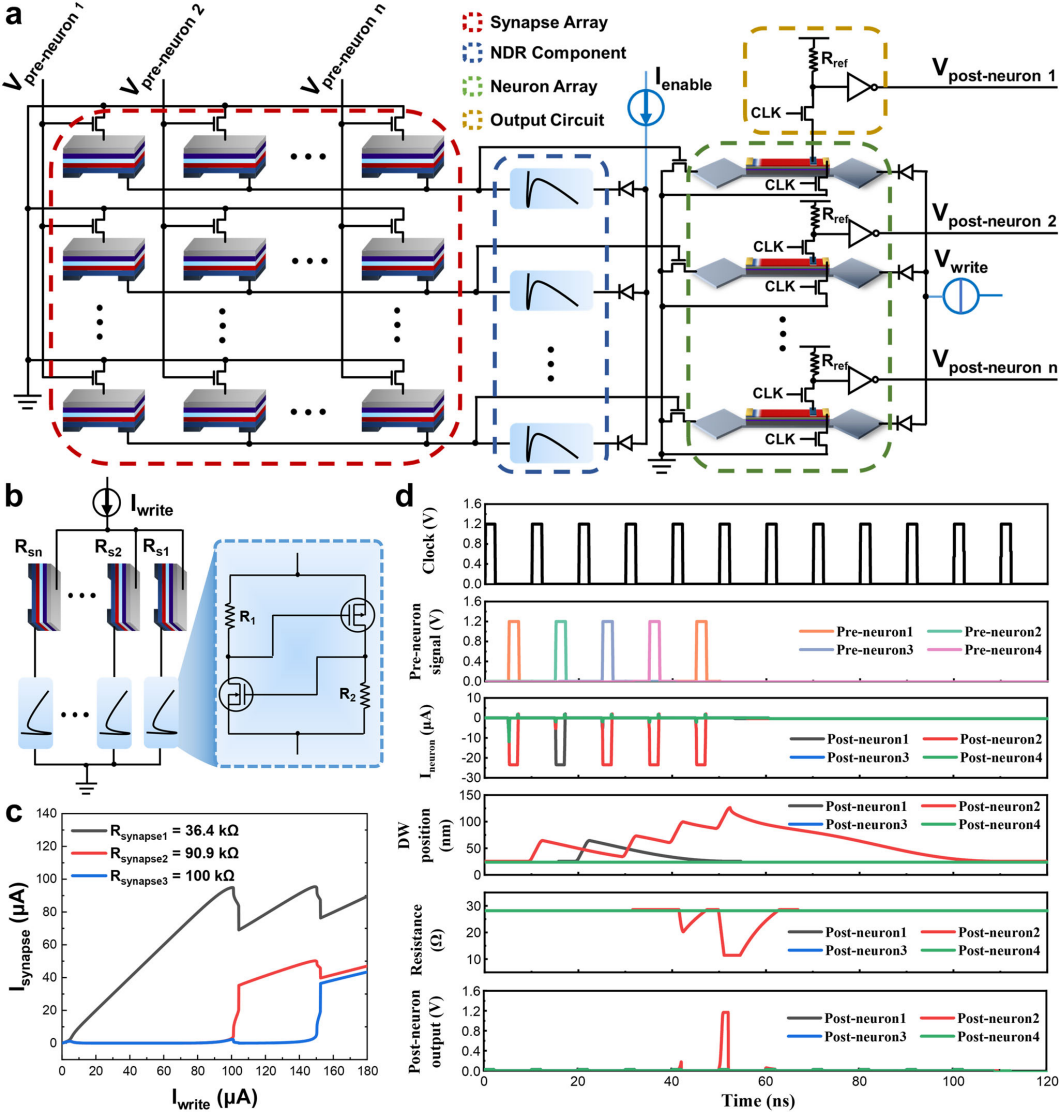
WTA with integrated negative differential resistance characteristics and spike neural circuit. **a** Basic structure of the spike neural circuit based on a synapses array, NDR devices array, LIF neurons array and output circuit. **b** Winner-takes-all neuron circuit combined with NDR devices. **c** Current differential characteristics of three branches with different synapse weights. **d** Transient simulation results.

The major roadblock to the development of large-scale neuron circuits is the high-power consumption. In a traditional neuron circuit, all neurons always produce an output for a given input, which causes the neurons to fire non-selectively and consume a lot of energy unnecessarily. In contrast, the biological neurons possess an intrinsic mechanism of lateral inhibition^61^, which ensures that only certain neurons can fire upon specific spiking events triggering. Therefore, to further reduce the power consumption of neuron devices in the neuron circuit in an optimized resource allocation way, a type of well-designed semiconductor components with NDR^40^ is integrated to realize the WTA function among our developed spintronic neurons. The writing current of neurons was precisely programmed upon the specific weights of the synapse array, as shown in Fig. 5a.

Motivated by the application of NDR in the previous report^62^, as depicted in Fig. 5b, I_enable_ refers to a constant current pulse of 35 μA input triggered by pre-neuron signals. Due to the different values of R_s1_, R_s2_,…, R_sn_, the current of each branch is different. The NDR device in which branch has the largest current will first enter into the negative differential resistance regime. As a result, the current in this branch will increase rapidly, while those in the other branches will decrease due to the drop of voltage at both ends of the circuit. In this way, the writing of a specific neuron device can be executed and the global inhibition among neurons is successfully achieved, which is more competitive than those devices with local inhibition WTA^11^. Importantly, the ratio of synaptic differential currents can exceed 10^4^, which makes the branch leakage current of those lose neurons to be negligible. The circuit diagram of the NDR component is illustrated in the inset of Fig. 5b, and its characteristics are shown in Supplementary Fig. S9. Representatively, Fig. 5c shows the current differential characteristics of three branches with different synapse weights.

Moreover, an SNN architecture based on the developed spintronic neurons and the integrated NDR WTA module was further implemented, as shown in Fig. 5a and schematically animated in Supplementary Movie 3. When the pre-neuron signal arrives, the reading control transistor of the synapse array is turned on and a constant current flow through the synapses array. Due to the different weights of synapses, the current flowing through each branch is different. Owing to the differential effect of the NDR devices, consequently, one neuron write control transistor is turned on, while the rest neurons associated transistors are turned off. Upon programmed pulses application from the pre-neurons, the WTA neuron will fire after a series of integrate and leaky actions, and generate a spike through the output circuit to realize the complete function of SNN. To make the simulation processes more efficient, a 4×4 crossbar consisting of four neuron devices was established with integrated NDR components. The implementation was performed by applying five current pulses with an amplitude of 23.5 μA, a duration of 2 ns and a period of 10 ns.

Briefly, the *pre-neuron1* signal arrived in the first cycle, and subsequently, the reading MOSFETs in the first column of the synaptic array were turned on instantly. Since the synapse connected to *neuron2* was in a low resistance state, *neuron2* won. Upon the response action from the NDR-WTA module, almost all portion of 23.5 μA current flew through *neuron2* and consequently inhibited the other neurons which guarantee negligible leakage current at a level of a few pA. Notably, *Neuron2* integrates in the first half of the period and the leakage process occurs in the second half. Similar to the first cycle, during the second period except *Neuron1* winning while *Neuron2* along with other neurons remain leaky. After five cycles, *Neuron2* eventually won and fired, i.e., realized the typical WTA behaviors, as illustrated in Fig. 5d, elaborating the feasibility and good circuit compatibility of developed spintronic neuron devices with neuron circuits implementation.

Compared to the latest representative WTA SNNs (Supplementary Table S5), our proposed scheme exhibits the competitive performance with 170 ps delay time and 90.99 μW overall power consumption, which is 3.4 × ~ 293× and 6.1× lower than that of the state-of-the-art, respectively^63,64^. Moreover, considering only the WTA module, the power consumption of our scheme is reduced by 21 × ~ 29× in comparison with the traditional WTA circuit^63,65^. Remarkably, as the network becomes more complex, the advantages of our design become more obvious. This will provide more possibilities for the application of high-speed, low-power and complex neuron circuits. Relatively long operating cycles can be optimized from a device perspective, such as by shortening the device length and thermal engineering.

### Spintronic LIFT-neuron-based SNN implementation

**Fig. 6:**
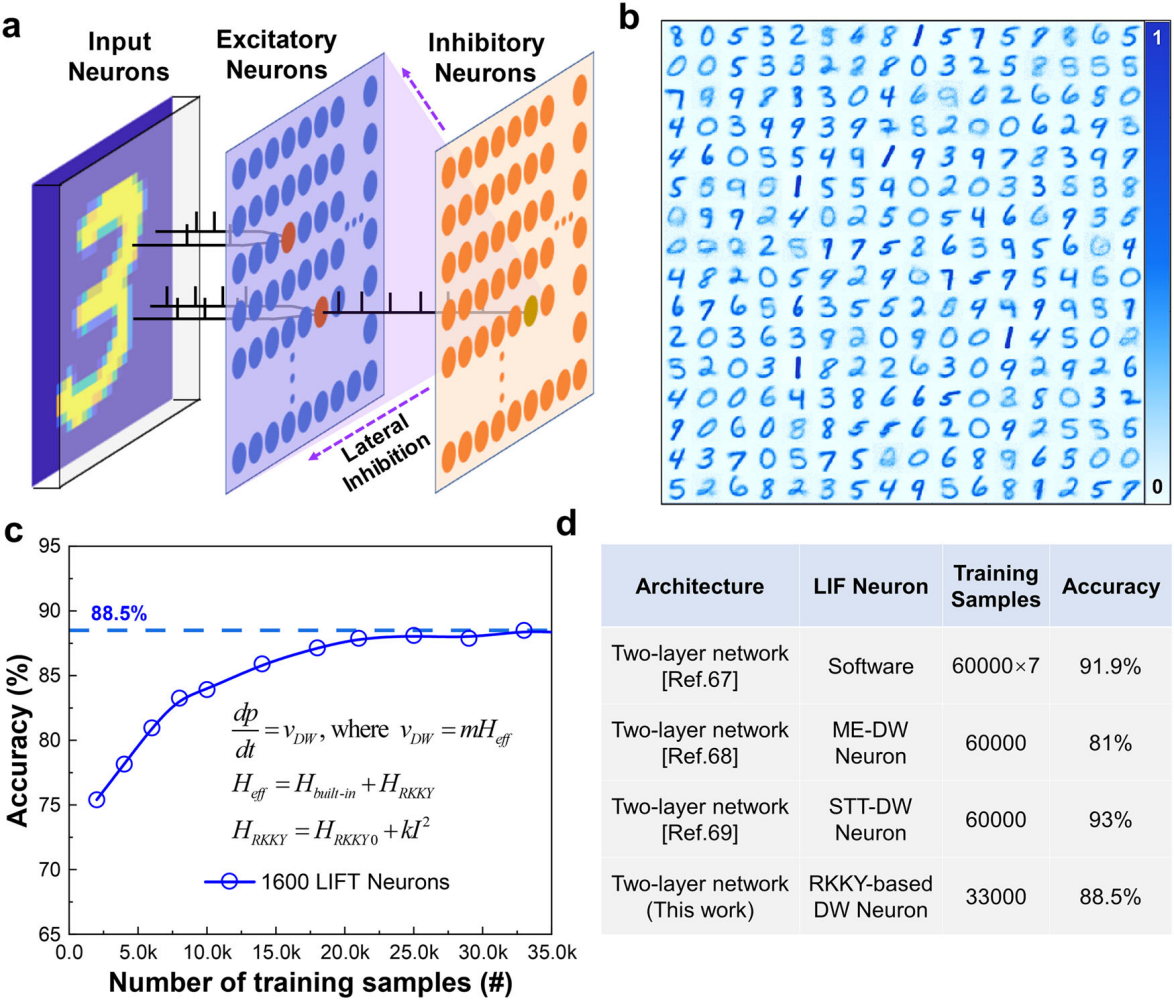
LIFT-SNN implementation. **a** LIFT-SNN topology structure. **b** Weight matrix of the first 256 excitatory neurons after training. **c** MNIST digital handwritten patterns recognition accuracy with 1600 excitation neurons under different number of training samples. **d** LIFT-SNN performance comparison of accuracy. ME Magnetoelectric.

From the application perspectives, the hardware implementations of neuromorphic computing are of paramount importance because of their competitive efficiency in solving recognition and classification tasks. We further evaluated the applicability of the developed spintronic LIFT neuron devices with implementation of typical Modified National Institute of Standards and Technology (MNIST) handwritten digits recognition. A spintronic LIFT neurons-based spiking neural network was constructed using an SNN simulator known as BRIAN2^66^. As shown in Fig. 6a, the topology of a typical two-layer SNN was adopted with inputs consisting of 2D array of 28×28 pixels. The Poisson encoding data flow was executed to trigger excitatory neurons via synapses thru spike-timing-dependent plasticity (STDP) learning. The input neurons are fully connected to the excitatory neurons and each excitatory neuron connects to a matching inhibitory neuron which inhibits the spiking event of all rest excitatory neurons^67^. The DW motion in each excitatory neuron device is regulated by the interconnected synaptic conductance, which calculates the value of the current flowing into the device, thereby generating Joule heating to drive the DW motion. In our device, the mechanism of effective magnetic field drives the magnetic DW motion can be expressed as the following physical model^45^, $v=m{H}_{eff}$, where $m=\gamma \varDelta /(\alpha+{\alpha }^{-1})$. *H*_eff_ is attributed to the competition between *H*_built-in_ and *H*_RKKY_, where H_RKKY_ is modulated by the Joule heating generated by the current, which can be written as the following model ${H}_{eff}={H}_{built-in}+{H}_{RKKY}$, ${H}_{RKKY}={H}_{RKKY0}+k{I}^{2}$, where *k* is the coefficient of Joule heating modulation of the RKKY field, which is extracted from our experimental results.

We further implemented an SNN with 1600 excitatory neurons and ten classes of handwritten digits, “0” through “9” were sequentially trained and inferenced. Figure 6b shows the representative first 256 excitatory neurons learned synaptic weights connecting the input neurons to excitatory neurons. Figure 6c depicts the handwritten patterns recognition accuracy with 1600 excitation neurons under different number of training samples after verification of 10,000 test sets. The average classification accuracy of ~88.5% was achieved with the first 33,000 samples of the training set for the SNN which learned the digits incrementally in a dynamic environment, demonstrating the comparable performance in comparison with the state-of-the-art^67–69^ as shown in the benchmark table in Fig. 6d. Note that the accuracy warrants to be enhanced further by optimizing model parameters, e.g., network structure, network size, and training parameters, etc.

## Discussion

Although the dynamic DW motion and modulation have been verified experimentally with neurons LIF featured behaviors, to further enhance the performance of the spintronic neuron devices, there is still a pressing need to carefully design the thermal heat generation and the CMOS compatible process technology. On the one hand, to improve the energy efficiency and eliminate the cross interference among neurons motivate researchers to simultaneously reduce the density of the local pinning center, i.e., improving the device reliability. In the large scale and high-density neuron circuits, thermal diffusion is a major problem to be resolved. On the other hand, our design may be more suitable for sparse and habitual neuron circuits^70,71^ by virtue of the nature of heat dissipation, which is also one of the focuses of our subsequent research, i.e., constructing neuron circuits with specific tasks. From the perspectives of material systems (e.g., FM/AFM/FM^72^, FM/FM(PM)/FM^73^), the Joule heating modulates not only the strength but also the sign of RKKY coupling, which is applicable for the CMOS devices integration. Furthermore, the voltage and strain bear extra engineering knob to modulate RKKY interaction^27,74,75^, enabling more reconfigurable neurons diversity. Importantly, the idea of introducing semiconductor negative differential resistance devices into SNN is expected to replace the traditional sense amplifier or analog-to-digital converter reading mode and further reduce the network overhead. Furthermore, the implemented two-layer SNN architecture achieves 88.5% accuracy on the MNIST benchmark under the unoptimized conditions, paving the way for more ambitious and futuristic applications of neuromorphic computing.

In conclusion, we have experimentally realized precise modulation of the RKKY effective field by Joule heating in a CMOS compatible SAF-structured neuron, and the spiking LIFT characteristics can be biologically emulated by the dynamic competition between RKKY interaction and H_built-in_. Such Joule heating-assisted DW-SAF spiking LIFT neuron requires neither the membrane capacitor nor the reset circuit because of its inherent RKKY-H_built-in_ competition property. Our developed spintronic spiking neurons demonstrated the LIF and self-reset functionalities with an ultra-low energy of 486 fJ/spike and a high firing rate up to 17 MHz, enabling attractive hardware acceleration compared to the biological counterpart. By integrating the NDR devices, the WTA functionalized among spintronic LIFT spiking neurons with a synaptic array current differential ratio >10^4^. An NDR-WTA spiking neuron circuit was successfully implemented with low latency of 170 ps and low power consumption of 90.99 μW. The proposed device-circuit codesign with synergistic tailoring of interlayer-coupling-induced magnetic DWM and integrating of NDR enables LIFT spiking neurons with WTA in neuron circuits, offering a reliable platform for future neuromorphic devices and chip applications. The established two-layer SNN based on our developed spintronic LIFT neurons that can be cascaded to the MTJ synapses crossbar with evading additional interfacing circuitry. The 88.5% accuracy has been achieved on the MNIST benchmark, facilitating the spintronic LIFT neurons hardware exploration for promising neuromorphic computing. It is believed that our studies will arouse a wide range of research in neuromorphic computing and pronounced interdisciplinarity, e.g., the CMOS integrated artificial neurons circuit tape-out process.

## Methods

### Film preparation and characterization

The films were deposited at room temperature onto high-resistivity Si/SiO_2_ wafers by DC and RF magnetron sputtering. Base pressure of ≤1 × 10^−6^ Pa and Ar gas were used during the sputtering. The epitaxial growth and crystal structure of SAF films stack were investigated by cross-sectional scanning transmission electron microscopy (STEM) analysis using FEI Titan Themis 200 TEM. The microscope was operated at 200 kV accelerating voltage. To improve STEM imaging quality and reduce the effects of sample drift and scanning noise, the method of drift corrected frame integration was implemented. The atomic level distribution of elements crossing the interfaces in the films stack were probed by secondary electron mass spectrometry (SIMS) using PHI nano TOF II TOF-SIMS (ULVAC-PHI).

### Device fabrication and measurement

The films were fabricated into Hall bar devices of 2 μm width and 50 μm length by optical lithography and ion beam etching. For DW velocity measurement, magnetic domain images were observed by MOKE microscopy of an MagVision system. Both the time interval and displacement of the DWs were extracted from the MOKE image and used to calculate the DW velocity. For electrical measurements, current was sourced by a Keithley 6221 and Hall voltage was measured by a Keithley 2182 A nanovoltmeter, both meters were controlled by MagVision system through scripts.

## Supplementary information

Supplementary Information

Description of Additional Supplementary Files

Supplementary Movie 1

Supplementary Movie 2

Supplementary Movie 3

## Data Availability

The datasets generated during and/or analyzed during the current study are available from the corresponding author on reasonable request.

## Code availability

The codes that support the findings of this study are available from the corresponding author upon reasonable request.
