# Supplementary material for: Spintronic leaky-integrate-fire spiking neurons with self-reset and winner-takes-all for neuromorphic computing
Source: Nat Commun. 2023 Feb 24;14:1068. doi: 10.1038/s41467-023-36728-1 (PMC9957988; doi:10.1038/s41467-023-36728-1)
Supplement: Supplementary file 1 — Supplementary Information [file 41467_2023_36728_MOESM1_ESM.pdf]

Supporting Information

Spintronic leaky-integrate-fire spiking neurons with self-reset and winner-takes-all for neuromorphic computing

Di Wang[1,2], Ruifeng Tang[1,2], Huai Lin[1,2], Long Liu[1,2], Nuo Xu[3], Yan Sun[4], Xuefeng Zhao[1,5], Ziwei Wang[1,2], Dandan Wang[6], Zhihong Mai[6], Yongjian Zhou[7], Nan Gao[5], Cheng Song[7], Lijun Zhu[8], Tom Wu[9], Ming Liu[1,2] and Guozhong Xing[1,2,5*]

[1]Key Laboratory of Microelectronics Devices & Integration Technology, Institute of Microelectronics, Chinese Academy of Sciences, Beijing, 100029, China

[2]University of Chinese Academy of Sciences, Beijing, 100049, China

[3]Department of Electrical Engineering and Computer Sciences, University of California, Berkeley, CA, 94720, USA

[4]Institute of Metal Research, Chinese Academy of Sciences, Shenyang 110016, China

[5]School of Microelectronics, University of Science and Technology of China, Hefei, Anhui 230026, China

[6]Jiufengshan Laboratory, Wuhan 430206 Hubei, China

[7]Key Laboratory of Advanced Materials (MOE), School of Materials Science and Engineering, Tsinghua University, Beijing 100084, China

[8]State Key Laboratory of Superlattices and Microstructures, Institute of Semiconductors, Chinese Academy of Sciences, Beijing, 100083, China

[9]Department of Applied Physics, The Hong Kong Polytechnic University, Kowloon, Hong Kong, China

*Corresponding author: Guozhong Xing (e-mail: gzxing@ime.ac.cn)

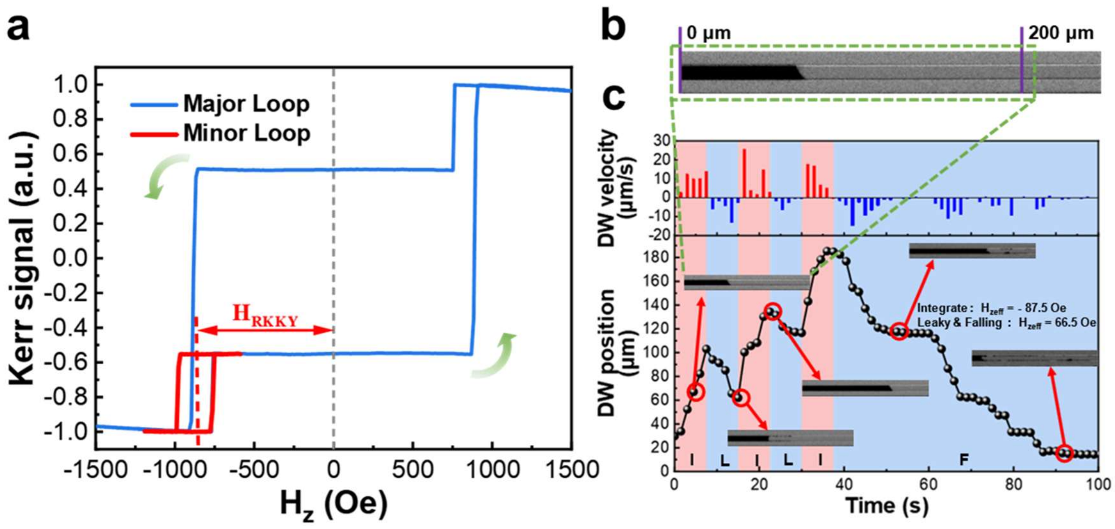

Figure S1 Magnetic field driven DW motion dynamics in patterned devices with SAF structure. **a** Major and minor K-H loops of the patterned device. H_{ex} refers to effective field from RKKY interaction. **b** Kerr images of the device recorded under a combined interlayer exchange coupling field H_{ex} and a constant external H_z field. **c** DW position as a function of time during integrate, leaky and falling processes. Pink regions depict the DW integrate process at effective OOP field H_z+H_{RKKY} = -87.5 Oe and the blue regions refer to DW leaky and falling processes at effective OOP field H_z+H_{RKKY} = 66.5 Oe. Kerr images illustrate the corresponding domain configuration in a timeframe. Upper histogram shows the DW velocity during the integrate, leaky and falling processes.

Parallelly, the results of realizing the LIF characteristics of neurons by driving DW motion with equivalent effective magnetic fields are shown in Supporting Information **Figure S1**. A series of strip devices were fabricated to investigate the dynamic processes of DW rising and falling. A constant negative B-field was applied and the DW was found to move rightward under an effective field of -87.5 Oe including the stray field and external field, emulating the process of rising. Subsequently, during the falling procession, the external field was reduced and the DW began to move to the left at 66.5 Oe effective field. The complete motion process of DW is shown in the Supporting Information **Figure S1b-c** with the corresponding Kerr images and DW velocity at the different moments as illustrated in the insets and the bar chart, respectively.

Note S1

To alleviate the DW motion stochasticity issue in the present work, the synergistic co-optimization approach was employed via engineering device fabrication process and tailoring LIFT neuron devices' operation mechanism.

✧ **Device fabrication process optimization**: The film surface/interface disorder and the roughness of device edge can be improved by optimizing the film deposition technology and device fabrication process[1-4]. In our work, besides securing the high-quality epitaxial growth of the films stack with post annealing treatment to reduce the intrinsic disorders and pinning effectively, the dedicated devices fabrication processes (i.e., the improved lithography with Al hard-mask and the optimized ion-beam etching with 30° tilting angle and rotation) were systematically executed to reduce the edge roughness and to minimize the imperfection sites, as verified by below tabulated extensive scanning electron microscopy (SEM) and atomic force microscopy (AFM) scanning images data with illustration of improved side-wall topology and edge roughness.

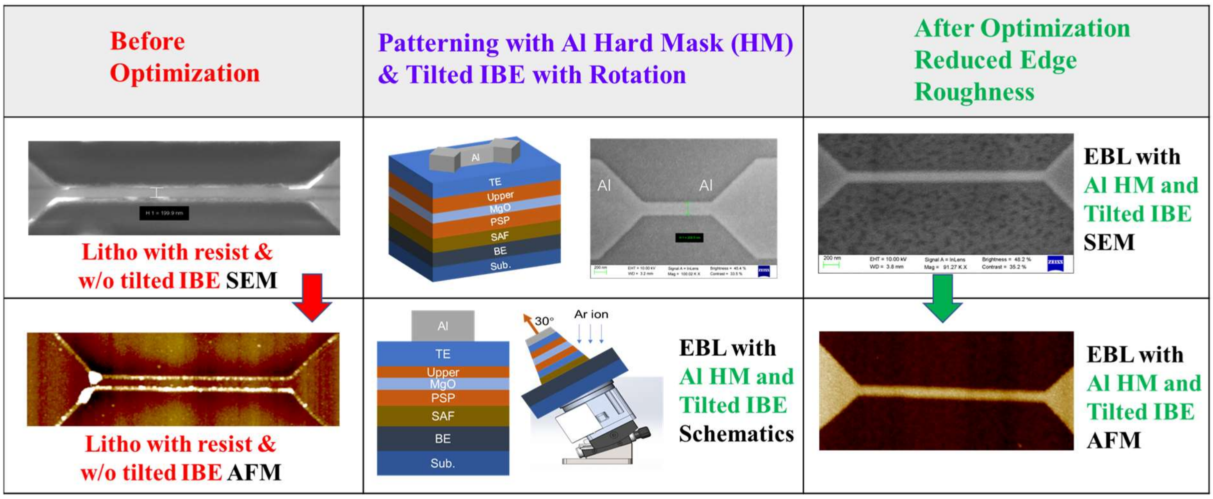

Figure S2. Typical SEM and AFM images data with the illustration of improved side-wall topology, morphology, and edge roughness, after devices fabrication engineering by using Al hard-mask (HM) and tilted ion-beam etching (IBE) process optimization.

✧ **Device mechanism and working principle**: Importantly, based on our device's working mechanism, the temperature increase caused by Joule heating is the inducement of DW motion, and the relationship between the pinning barrier and temperature satisfies the Kurkijärvi model [i.e., $H_{\mathrm{depin}}(T)=H_0\left\{1-\left[\frac{k_BT}{E_0}\ln\left(\frac{\Gamma_0 k_B T H_0}{1.5E_0 \upsilon\sqrt{1-H_{\mathrm{depin}}/H_0}}\right)\right]^{2/3}\right\}$, the temperature dependence of the depinning field per

reports[5, 6], and the pinning field decreases with increasing temperature simultaneously. This allows domain walls to maintain consistent magnetization structures during propagation, fundamentally plays important role in harnessing the DW motion stochasticity issue, in turn facilitating the SNN implementation with competitive digit recognition accuracy and performance.

Upon synergistic co-optimization with the maximized effective field acting on the DW, so that the DW motion falls closer to the flow regime. As shown in **Figure S3**, the phenomenon of stochastic DW motion has been significantly improved.

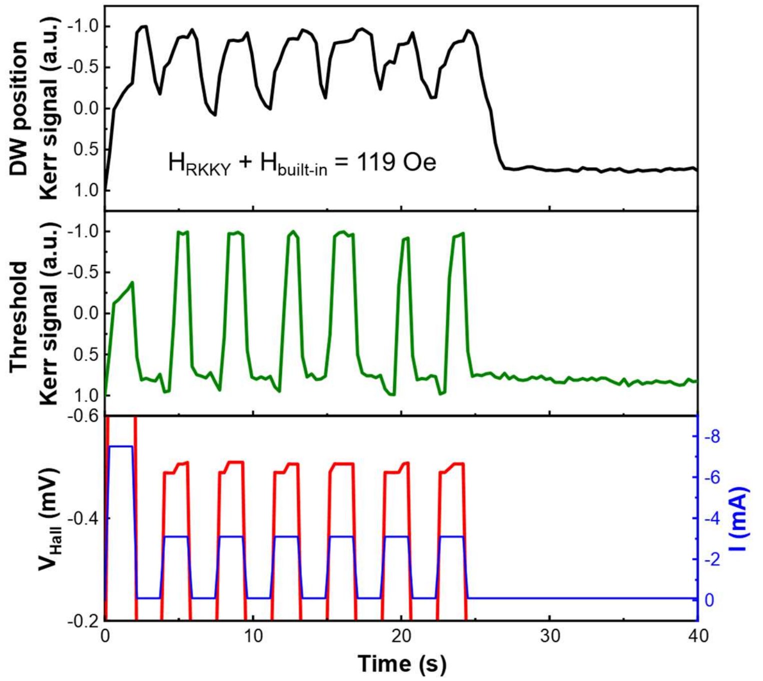

Figure S3. Extensive observation of dynamic DW motion emulated LIFT processes upon synergistic co-optimization with a maximized effective field acting on the DW, complementarily facilitating our established spintronic LIFT neuron model for spiking neural network implementation.

Note S2

The extensive experiments of RKKY effective field extraction were performed as a function of the current with 25 repetitions on each single data point. The field induced by non-Joule-heating-induced-field (current-induced field, CIF) can be calculated by the modulated RKKY effective field at different positive and negative currents using the following equations,

$$\begin{cases} JHIF = \left(H_{RKKY}\left(+I\right) + H_{RKKY}\left(-I\right)\right)/2 \\ CIF = \left(H_{RKKY}\left(+I\right) - H_{RKKY}\left(-I\right)\right)/2 \end{cases}$$

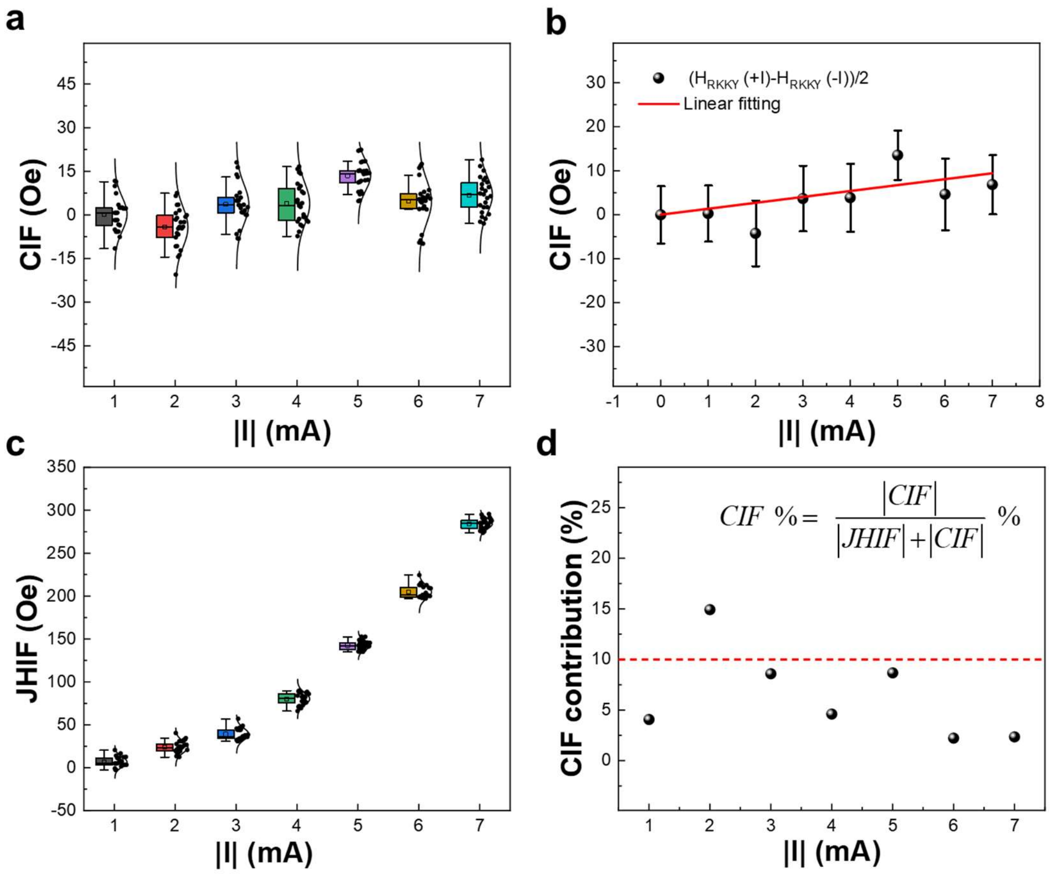

Figure S4. The dependence and contribution of the effective field induced by current polarity.

Therefore, we calculated CIF and plotted the relationship between CIF and current amplitude, as shown in **Figure S4**. The dependence of the effective field polarity on the current shows an approximate linear relationship, about 1.34 Oe/mA, which is quite limited in our experiment. As manifested in the **Figure 3b**, with less than 5 Oe the non-Joule-heating-induced rider field strength is <10% portion of JHIF. Clearly, as depicted in **Figure 3b**, the JHIF is dominated in modulation of the RKKY interaction, corroborated by our reproducible experiments.

Supporting Information Table S1 COMSOL simulation parameters.

**Material**	**κ [W/(m•K)]**	**ρ[10^3 kg/m^3]**	**C_p[J/(kg•K)]**
Ta	57	16.7	140
Ru	117	12.4	238
Pt	71.6	21.5	133
W	174	19.4	132
MgO	4	3.6	935
SiO_2	1.4	2.2	1052
Co	100	8.9	420
CoFeB	87	8.2	440
ScN	54	4.4	687

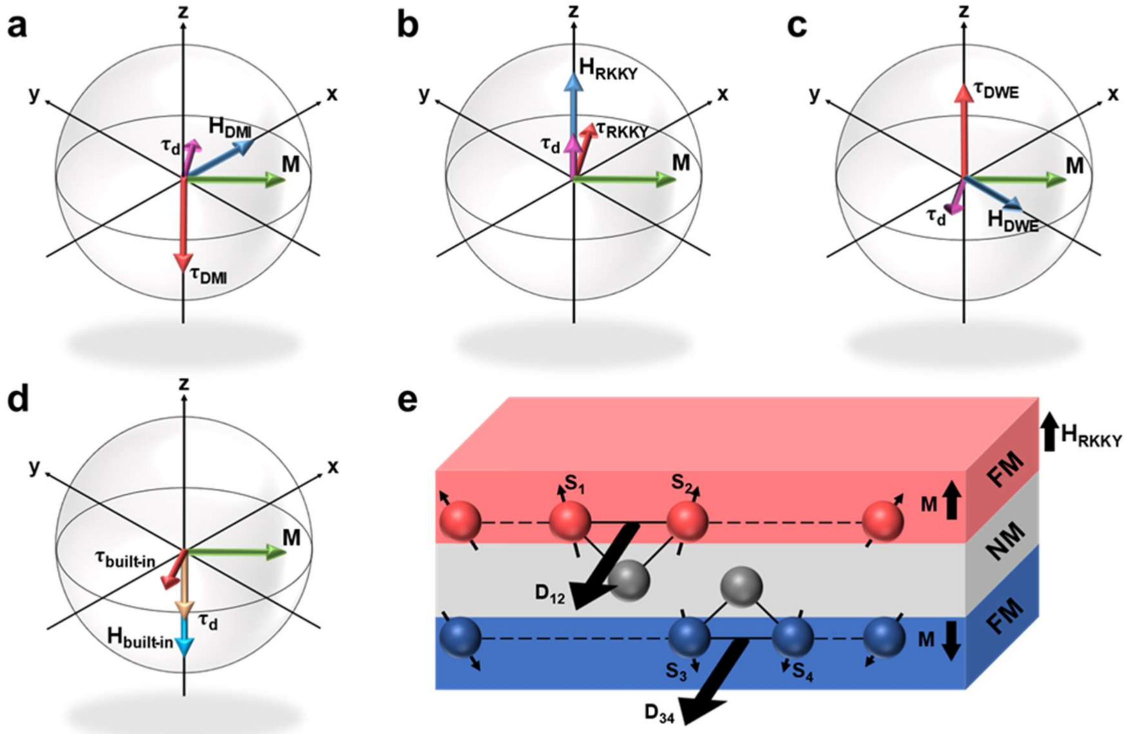

Figure S5 Schematic illustration of proposed artificial neuron device with synergistic driving torques and Dzyaloshinskii–Moriya interaction. a-d Directions of fields and torques shown schematically for upper ferromagnetic layer of SAF. **M** is the magnetizations in the center of the DW, the longitudinal torque $\boldsymbol{\tau}_{\mathbf{DMI}}$ is derived from longitudinal fields $\mathbf{H}_{\mathbf{DMI}}$ that are mainly contributed by the corresponding DMI field, the exchange torque $\boldsymbol{\tau}_{\mathbf{RKKY}}$ is derived from the antiferromagnetic exchange coupling field $\mathbf{H}_{\mathbf{RKKY}}$ in SAF, the domain wall energy torque $\boldsymbol{\tau}_{\mathbf{DWE}}$ is derived from domain wall energy fields $\mathbf{H}_{\mathbf{DWE}}$ that are mainly contributed by the corresponding magnetic anisotropic field, the built-in torque $\boldsymbol{\tau}_{\mathbf{built\text{-}in}}$ is derived from built-in fields $\mathbf{H}_{\mathbf{built\text{-}in}}$ and the damping torque $\boldsymbol{\tau}_{\mathbf{d}}$ is induced by the motion $\frac{\mathrm{dm}}{\mathrm{dt}}$. **e** At the interface between ferromagnet and non-magnetic metals (especially heavy metals), due to the lack of inversion center, two magnetic atoms will undergo spin orbit coupling with a third non-magnetic atom, resulting in DMI, which can be expressed as $\mathbf{-D_{12}\cdot(S_1\times S_2)}$, where $\mathbf{D_{12}}$ is the Dzyaloshinskii vector. $\mathbf{H_{ex}}$ is the antiferromagnetic exchange coupling field of lower FM acting on upper FM.

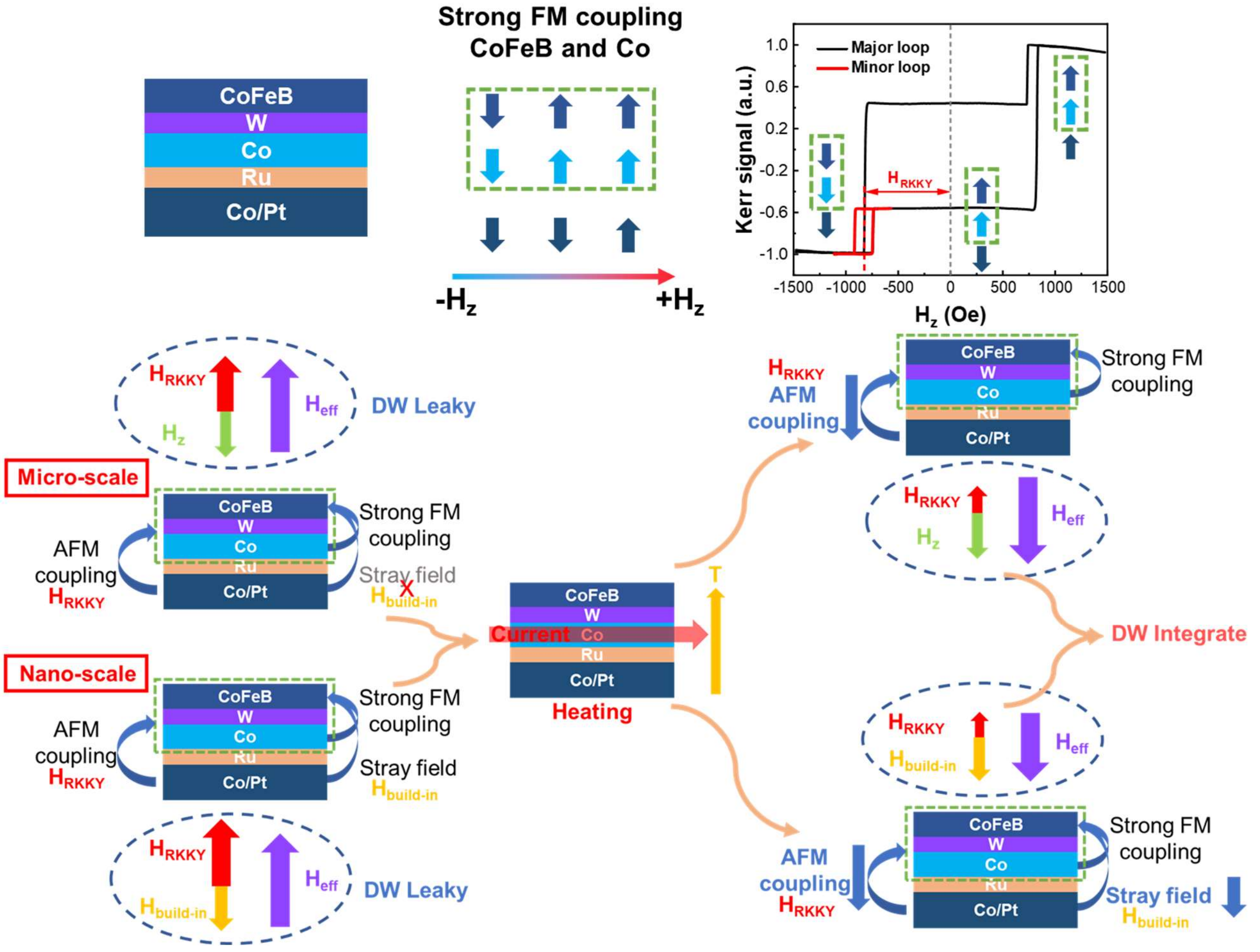

Figure S6 Schematic model of Joule Heat modulated RKKY interaction in the tailored SAF heterostructure.

As specifically elaborated in the Supporting Information **Figure S6**, a schematic model of Joule heating modulated RKKY interaction is discussed in detail with insights into the DW motion dynamics. By modulating the thickness of the spacer layer in the stacked structure samples prepared in our experiments, the films with different RKKY coupling strength can be adjusted precisely. In our samples, the strong FM coupling between perpendicularly spin-polarized CoFeB and Co leads to a coherent magnetization switching. When the field is swept from the negative saturation to the positive saturation, the CoFeB and Co ferro-coupled magnetization switches synchronously first due to the strong RKKY interaction. When the external field is large enough, the Co/Pt multilayer also switches, and finally the hysteresis loop presents a triple loop as shown in **Figure 1f** in the main text.

Based on our experimental results and simulation studies, the plausible mechanism of DW motion driven by competition between the intrinsic built-in field (i.e., stray field) and RKKY field in the thin films stack is discussed in detail. In the micron-scale device in our experiment, the internal

stray field is much smaller than the RKKY field and can be negligible[7, 8]. Correspondingly, a relatively weak external magnetic field is applied with opposite polarity to RKKY field. As a result, the effective field subjected to DW in CoFeB and Co layers is positive, which can drive the down-up DW to move to the left, emulating the neuron leaky process. When the device is manipulated by the Joule heating generated via a pulsed current flow, the RKKY coupling decreases linearly with increasing temperature. Consequently, the amplitude of RKKY effective field is smaller than the external H_z field, and the net effective field is negative, leading to DW moving rightward and mimicking the characteristics of neuron integrate in a biological manner. However, when the device is shrink to nanoscale size, the strong stray field inside the SAF film[8] with opposite polarity plays a critical role in competing the RKKY field, elaborating the same functions as micro-scale samples.

The Joule heating induced RKKY modulation is ascribed to the following mechanisms[9]:

- Spacer contribution

The interlayer-exchange-coupling is reduced due to the softening of the Fermi edge at higher temperature.

$$f^{spacer}(D,T) = f(D \cdot T)$$

- Interface contribution

The complex reflection coefficients at the spacer/magnet interface is highly energy dependent, which results in a strong dependence on temperature.

$$f^{interface}(D,T) = f(T)$$

- Magnetic layers

Collective excitation within the magnetic layers will reduce the free energy of the system.

$$f^{magnet}(D,T) = f(J_{inter}(0,D),T)$$

The overall influence of the above factors on RKKY interaction can be written as:

$$f(T) = \frac{J_{inter}(T)}{J_{inter}(0)} = \frac{cT}{\sinh(cT)}$$

Approximately, within a certain temperature range, the following relationship is derived as follows:

$$\delta J_{RKKY} \propto \delta T$$

The basic dynamic process of DWs and corresponding simulation parameters are shown in Supporting Information **Figure S7** and **Table S2**, respectively.

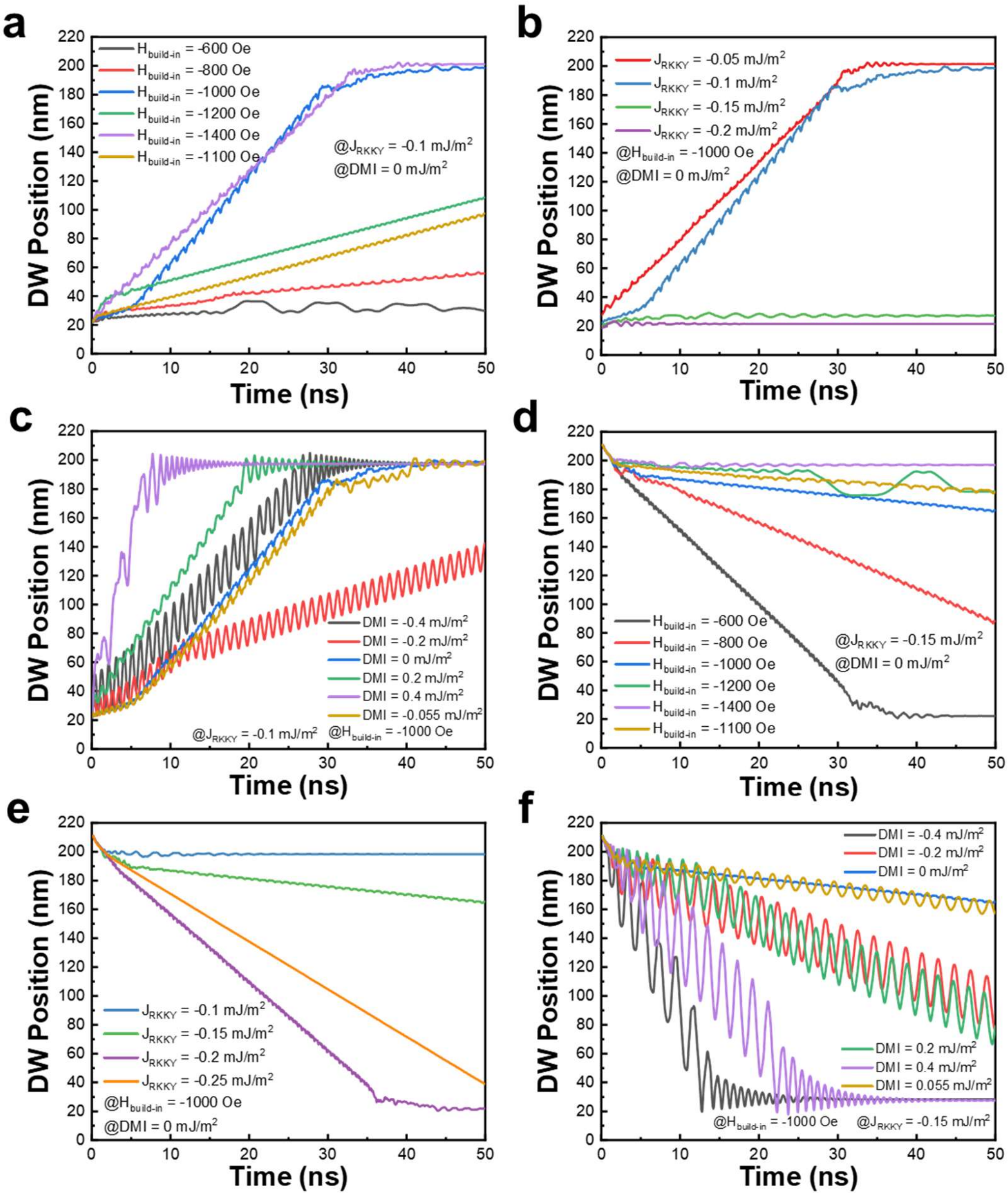

Figure S7 Characteristics of proposed LIF neuron devices under various physical parameters. **a** Integrate behavior of neuron device with different built-in field, DMI = 0 mJ/m^2 and J_{RKKY} = -0.1 mJ/m^2. **b** Integrate behavior of neuron device with different RKKY interaction strength, $H_{built\text{-}in}$ = -1000 Oe and DMI = 0 mJ/m^2. **c** Integrate behavior of neuron device with different DMI strength, J_{RKKY} = -0.1 mJ/m^2 and $H_{built\text{-}in}$ = -1000 Oe. **d** Leaky behavior of neuron device with different RKKY interaction strength, DMI = 0 mJ/m^2 and J_{RKKY} = -0.15 mJ/m^2. **e** Leaky behavior of neuron device with different DMI strength, $H_{built\text{-}in}$ = -1000 Oe and DMI = 0 mJ/m^2. **f** Leaky behavior of neuron device with different DMI strength, J_{RKKY} = -0.15 mJ/m^2 and $H_{built\text{-}in}$ = -1000 Oe.

Supporting Information Table S2 Micromagnetic simulation parameters.

Parameters	Description	Values
M_s	Saturation magnetization	1×10^{6} A/m
K_u	Effective magnetic anisotropy	8×10^{5} J/m^{3}
A	Exchange stiffness constant	1×10^{-11} J/m
α	Gilbert damping constant	0.02
DMI	Dzyaloshinskii-Moriya interaction strength	-0.4 to 0.4 mJ/m^{2}
J_{RKKY}	Ruderman-Kittel-Kasuya-Yosida exchange strength	-0.05 to -0.2 mJ/m^{2}
$H_{Built\text{-}in}$	Built-in field	-600 to -1400 Oe
t_p	Pulse width	2 ns
$(L\times W\times t)_{AFM}$	Boundary antiferromagnetic pinning layer dimension	$10\times50\times1$ nm^{3}
$(L\times W\times t)_{FL}$	Free layer dimension	$220\times50\times1$ nm^{3}

Supporting Information Table S3 Comparison and benchmark among different typical LIF neuron devices.

Category		CMOS[10]	GeSbTe[11]	NbO_x[12]	HZO-FeFET[13]	DW-MTJ[14]	T-SOT-HB[15]		This work
Neuron type		LIF	Stochastic/ IF	LIF	LIF	LIF	Probability/LIF		LIF
Area/Size		0.01 mm^2	100×100 nm^2	25 μm^2	6400 μm^2	600×32 nm^2	5 μm	100 nm	220×50 nm^2
Membrane capacitor		Yes	No	Yes	No	No	No	No	No
Raising time		-	-	-	~5 μs	~6.5 ns	-	-	10 ns
Falling time		-	-	461 ns	~200 μs	~220 ns	-	-	50 ns
Reset circuit		Yes	Yes	Yes	Yes	No	No	No	No
Operation period	Integrate	0.5 μs	50 ns	1 μs	1 μs	2 ns	175 ns	~ns	2 ns
	Leaky	-	-	0.1 μs	1 μs	30 ns	-	-	8 ns
Firing rate		-	5-15 kHz	~MHz	1.2 Hz	< 4.5 MHz	<10 MHz	-	0.017~17M Hz
Energy per spike		9.3 pJ	5 pJ	>431 pJ	>0.64 nJ	368.6 fJ	70 nJ	sub-pJ	486 fJ
Integrate power (μW)		18.6	100	Average: 392	>640	184.3	4×10^5	<1000	243
Investigations methodology		Experiment	Experiment	Experiment	Experiment	Simulation	Experiment	Calculation	Exp. & Simu.

Note S3

To comprehensively demonstrate the performance of the device, we have listed the calculations of power and energy consumption of our device and the data extracted from other highly relevant reports.

GeSbTe[11]

$$E = 5\ \text{pJ/spike}$$

$$P = \frac{E}{t_p} = \frac{5\text{pJ}}{50\text{ns}} = 100\ \mu\text{W}$$

HZO-FeFET[13]

$$E = \frac{CV^2}{2} = \frac{1.28\text{nF}\times 1\text{V}^2}{2} = 0.64\ \text{nJ/spike}$$

$$P = \frac{E}{t_p} = \frac{0.64\text{nJ}}{1\mu\text{s}} = 640\ \mu\text{W}$$

DW-MTJ[14]

$$P = I^2R = \frac{I^2\rho l}{wh} = (2\times10^8\text{A/cm}^2\times32\times10^{-9}\text{m}\times1.5\times10^{-9}\text{m})^2\times(160\mu\Omega\cdot\text{cm}\times600\times10^{-9}\text{m}\div32\times10^{-9}\text{m}\div1.5\times10^{-9}\text{m})$$

$$= 184.3\ \mu\text{W}$$

$$E = P\times t_p = 184.3\mu\text{W}\times2\text{ns} = 368.6\ \text{fJ/spike}$$

This Work

$$P = I^2R = \frac{I^2\rho l}{wh} = (23.5\times10^{-6}\text{A})^2\times(200\text{m}\Omega\cdot\text{cm}\times220\text{nm}\div50\text{nm}\div2\text{nm}) = 243\ \mu\text{W}$$

$$E = P\times t_p = 243\mu\text{W}\times2\text{ns} = 486\ \text{fJ/spike}$$

Based on the micromagnetic simulation results, an effective Verilog-A behavioral model was established for co-simulation with the CMOS circuit and the details of the mathematic model are shown in Supporting Information **Note 2** and **Figure S8**. The relevant parameters are listed in Supporting Information **Table S4**.

Note S4

MTJ Model

The parallel resistance of MTJ can be expressed by Brinkman model[16]:

$$R_P = \frac{t_{\mathrm{ox}}}{FA\sqrt{\varphi_{\mathrm{ox}}}WL} e^{\left(\frac{2\sqrt{2m|e|\varphi_{ox}}t_{ox}}{\hbar}\right)};$$

where φ_{ox} is the potential barrier height of MgO and m is the electron mass. FA is a fitting coefficient, calculated by $FA = 3.3141 \times 10^{-7} / RA$, where RA is the resistance–area product.

When in a fully antiparallel state, the resistance of MTJ is described by the following equation[16]:

$$R_{AP} = R_P\left(1 + TMR\right);$$

where TMR is the tunneling magnetoresistance ratio.

As a result, the resistance of MTJ can be expressed as:

$$R_{MTJ}(x) = \begin{cases} R_{AP}, & x < x_{MTJ} - \frac{l_{MTJ}}{2} \\ \frac{R_P\left(x - x_{MTJ} + l_{MTJ}\right)}{L} + \frac{R_{AP}\left(x_{MTJ} + l_{MTJ} - x\right)}{L}, & x_{MTJ} - \frac{l_{MTJ}}{2} < x < x_{MTJ} + \frac{l_{MTJ}}{2} \\ R_P, & x > x_{MTJ} + \frac{l_{MTJ}}{2} \end{cases}$$

where x_{MTJ} is the location of the center of MTJ and l_{MTJ} is the length of MTJ.

Mathematic model of DW motion:

The process of DW falling is nonlinear with position due to the dynamic RKKY interaction. Therefore, a polynomial scheme involves only non-negative integer powers or only positive integer exponents of a variable is applied to fit the results of micromagnetic simulations to obtain the specific expression of DW velocity as function of the time and the position.

Figure S8

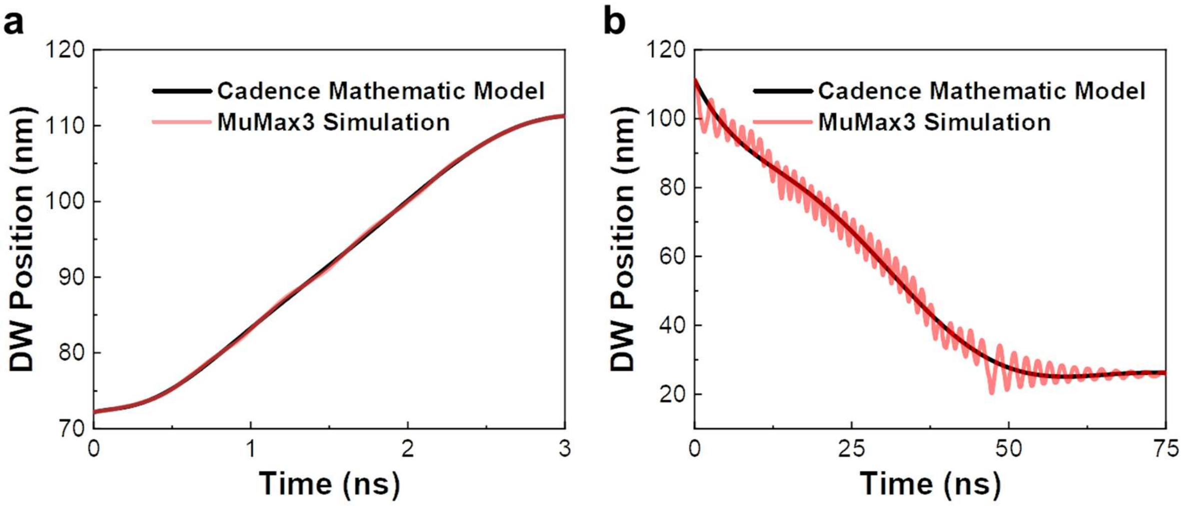

Figure S8 DW integrate and leaky process in MuMax3 simulation and Verilog-A mathematical model.

Table S4

Supporting Information Table S4 Cadence simulation parameters.

Parameters	Description	Values
V_{dd}	Supply voltage	1.2 V
V_{write}	Writing voltage	11.5 V
T	Clock period	6 ns
ω	Clock pulse width	3 ns
L_{MOS}	Channel length of MOSFET	60 nm
TMR	Tunnel magnetoresistance ratio	150%
I_{enable}	Enable differential current	35 μA
t_p	Pulse width	3 ns
R_{ref}	Reference resistance of the reading circuit	40 kΩ
R_{ps}	Low resistance value of synapse MTJ	36.4 kΩ
ρ_{FL}	Free layer (CoFeB) resistivity	80 μΩ·cm
ρ_{SL}	Spacer layer (β-W) resistivity	250 μΩ·cm
ρ_{Heater}	Heater (Sc,Mn)N resistivity	200 mΩ·cm
t_{ox}	Thickness of barrier layer	1.2 nm
R_{pn}	Low resistance value of neuron detecting MTJ	17.1 kΩ
$(L\times W)_{MTJ}$	Neuron detecting MTJ dimension	20×50 nm^2
$(L\times W\times t)_{AFM}$	Boundary antiferromagnetic pinning layer dimension	10×50×1 nm^3
$(L\times W\times t)_{SL}$	Spacer layer dimension	220×50×0.5 nm^3
$(L\times W\times t)_{FL}$	Free layer dimension	220×50×1 nm^3
$(L\times W\times t)_{Heater}$	Heater dimension	220×50×20 nm^3

Figure S9

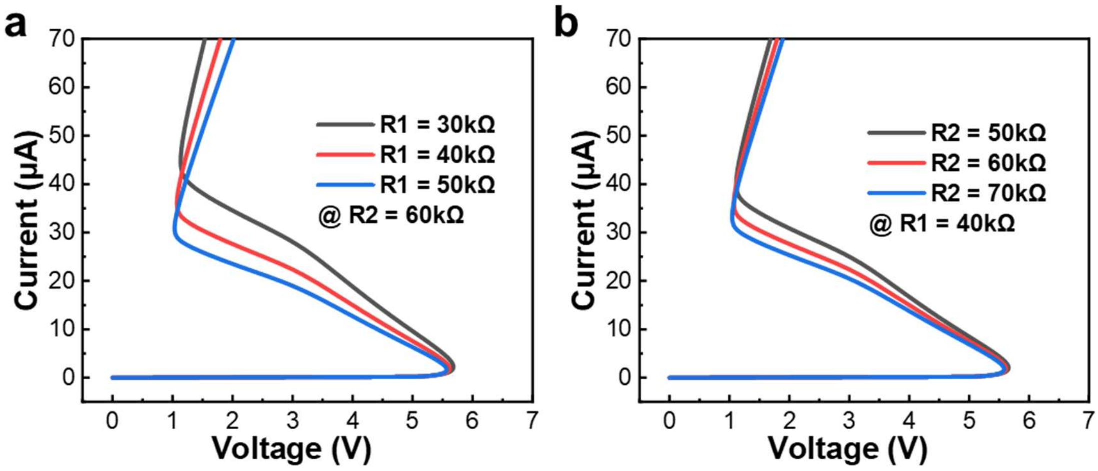

Figure S9 Electrical characteristics of the NDR component. a Voltage is a function of current at different R1. **b** Voltage is a function of current at different R2.

Table S5 Benchmark of latest representative WTA SNNs including the one from the current work.

Category		SR-WTA[17]	CMOS WTA[18]	CMOS WTA[19]	This work	
**Number of neurons**		3×1	10×1	8×1	4×1	
**Delay (ns)**		0.75	50	12 (8)	0.17	
**Period (ns)**		2	-	-	10	
**Power consumption (μW)**	**Synapse array**	650	-	-	90.99	9.4
	**Neuron array**		-	-		52.6
	**NDR-WTA**		1200 (120/cell)	87.5/cell		16.0
	**Reading**		-	-		13.3

*SR-WTA: Skyrmion Racing Winner-Takes-All.

**Delay of CMOS WTA[19]: Outside the parentheses is simulation result, and inside is test result.

References

1. Van de Wiele B, Laurson L, Durin G. Effect of disorder on transverse domain wall dynamics in magnetic nanostrips. Phys Rev B **86**, 144415 (2012).
2. Chen TY, Erickson MJ, Crowell PA, Leighton C. Surface Roughness Dominated Pinning Mechanism of Magnetic Vortices in Soft Ferromagnetic Films. Phys Rev Lett **109**, 097202 (2012).
3. Albert M, Franchin M, Fischbacher T, Meier G, Fangohr H. Domain wall motion in perpendicular anisotropy nanowires with edge roughness. Journal of Physics-Condensed Matter **24**, 024219 (2012).
4. Nakatani Y, Thiaville A, Miltat J. Faster magnetic walls in rough wires. Nat Mater **2**, 521-523 (2003).
5. Kurkijärvi J. Intrinsic Fluctuations in a Superconducting Ring Closed with a Josephson Junction. Phys Rev B **6**, 832-835 (1972).
6. Lendecke P, Eiselt R, Meier G, Merkt U. Temperature dependence of domain-wall depinning fields in constricted Permalloy nanowires. J Appl Phys **103**, 073909 (2008).
7. Bandiera S, et al. Comparison of synthetic antiferromagnets and hard ferromagnets as reference layer in magnetic tunnel junctions with perpendicular magnetic anisotropy. IEEE Magn Lett **1**, 3000204-3000204 (2010).
8. Baltz V, Rodmacq B, Bollero A, Ferre J, Landis S, Dieny B. Balancing interlayer dipolar interactions in multilevel patterned media with out-of-plane magnetic anisotropy. Appl Phys Lett **94**, 052503 (2009).
9. Schwieger S, Nolting W. Origin of the temperature dependence of interlayer exchange coupling in metallic trilayers. Phys Rev B **69**, 224413 (2004).
10. Wu XY, Saxena V, Zhu KH, Balagopal S. A CMOS spiking neuron for brain-inspired neural networks with resistive synapses and in situ learning. IEEE Trans Circuits Syst II Express Briefs **62**, 1088-1092 (2015).
11. Tuma T, Pantazi A, Le Gallo M, Sebastian A, Eleftheriou E. Stochastic phase-change neurons. Nat Nanotechnol **11**, 693-699 (2016).
12. Duan Q, et al. Spiking neurons with spatiotemporal dynamics and gain modulation for monolithically integrated memristive neural networks. Nat Commun **11**, 3399 (2020).
13. Dutta S, et al. Biologically Plausible Ferroelectric Quasi-Leaky Integrate and Fire Neuron. In: 2019 Symposium on VLSI Technology). IEEE (2019).
14. Hassan N, et al. Magnetic domain wall neuron with lateral inhibition. J Appl Phys **124**, 152127 (2018).
15. Kurenkov A, DuttaGupta S, Zhang C, Fukami S, Horio Y, Ohno H. Artificial neuron and synapse realized in an antiferromagnet/ferromagnet heterostructure using dynamics of spin–orbit torque switching. Adv Mater **31**, 1900636 (2019).
16. Wang C, Wang Z, Wang M, Zhang X, Zhan Y, Zhao W. Compact model of Dzyaloshinskii domain wall motion-based MTJ for spin neural networks. IEEE Trans Electron Devices **67**, 2621-2626 (2020).
17. Pan B, et al. SR-WTA: Skyrmion racing winner-takes-all module for spiking neural computing. In: 2019 IEEE International Symposium on Circuits and Systems (ISCAS)). IEEE (2019).

18. Choi J, Sheu BJ. A high-precision VLSI winner-take-all circuit for self-organizing neural networks. IEEE J Solid-State Circuits **28**, 576-584 (1993).
19. Fish A, Milrud V, Yadid-Pecht O. High-speed and high-precision current winner-take-all circuit. IEEE Trans Circuits Syst II Express Briefs **52**, 131-135 (2005).
