# Supplementary material for: Spintronic leaky-integrate-fire spiking neurons with self-reset and winner-takes-all for neuromorphic computing
Source: Nat Commun. 2023 Feb 24;14:1068. doi: 10.1038/s41467-023-36728-1 (PMC9957988; doi:10.1038/s41467-023-36728-1)
Supplement: Supplementary file 2 — Description of Additional Supplementary Files [file 41467_2023_36728_MOESM2_ESM.docx]

**Supplementary Data Legends**

**Supplementary Movie 1** DW-based spintronic neuron LIF and self-reset (LIFT) process in the developed devices.

**Supplementary Movie 2** Simulated LIFT process in the proposed nanoscale spintronic neurons.

**Supplementary Movie 3** WTA-SNN implementation demonstration based on the developed spintronic LIFT neurons with integration of NDR devices.
